# Machine Learning for Gas Capture in Ionic Liquids: Current Status and Future Trends

**DOI:** 10.3390/molecules31132293

**Published:** 2026-07-01

**Authors:** Guocai Tian, Zhiqiang Hu, Ranran Geng

**Affiliations:** State Key Laboratory of Complex Non-Ferrous Metal Resource Clean Utilization, Faculty of Metallurgical and Energy Engineering, Kunming University of Science and Technology, Kunming 650093, China

**Keywords:** ionic liquids, green solvents, QSAR, machine learning, gas capture, solubility

## Abstract

Ionic liquids, as green gas solubility media, have great potential for applications in carbon capture, industrial waste gas purification, and other fields. However, the massive combination of anions and cations makes their screening extremely difficult. Machine learning can break through the bottleneck of traditional experiments and simulations and achieve high-throughput prediction of gas solubility in ionic liquids. This article provides a systematic review of the research progress of machine learning in predicting the gas solubility performance of ionic liquids. The classification and modeling process of machine learning, the construction and performance of machine learning prediction models for the solubility of gases such as CO_2_, H_2_S, NH_3_, SO_2_, N_2_O and others in ionic liquids were analyzed and summarized. The progress and existing problems of machine learning application for gas capture in ionic liquids and the future development direction are discussed, in order to provide assistance and theoretical reference for the directional design and industrial application of ionic liquids.

## 1. Introduction

Gas separation and absorption are core unit operations in the fields of chemical, energy, and environmental engineering, widely used in key scenarios such as carbon capture and storage (CCS), coal-fired flue gas desulfurization and denitrification, acid gas purification in oil and gas fields, and industrial volatile organic compound treatment [1,2,3,4,5]. With the deepening of the global “dual carbon” strategy and the upgrading of industrial green transformation, efficient, green, and low-cost gas capture and separation technology has become a hot research topic in the industry. Traditional gas absorbents mainly include water, alcohol, amine organic solvents, carbonate solutions, etc., which generally have problems such as high volatility, corrosion of equipment, high regeneration energy consumption, poor selectivity, and easy secondary pollution, greatly limiting the energy-saving and green development of gas separation processes [6].

Ionic liquids are room temperature molten salts composed of larger organic cations and some smaller organic/inorganic anions, which are liquid at or near room temperature [7,8,9,10,11,12,13]. With unique advantages such as almost good chemical stability, zero vapor pressure, excellent thermal stability, designable structure, strong gas solubility, and high selectivity, ionic liquids perfectly avoid the technical shortcomings of traditional absorbents and become a new generation of green gas capture media to replace traditional organic solvents [14]. By directional modification and combination design of functional groups of cations and anions in ionic liquids, their adsorption affinity and dissolution capacity for different gases can be precisely controlled, achieving efficient selective capture of gases. This has broad industrial application prospects in the field of industrial gas purification [15,16,17,18,19]. In recent years, ionic liquids have made significant progress in gas capture and separation of CO_2_ [20,21,22], SO_2_ [23,24], H_2_S [25,26], NH_3_ [27,28], and other gases.

In the process of gas separation and absorption, gas solubility is the core basic data for evaluating the gas solubility performance of ionic liquids, designing absorption process parameters, and optimizing equipment structure. Accurately obtaining gas solubility data at different temperatures, pressures, and ionic liquid structures is a prerequisite for the industrial application of ionic liquids. Due to the combination of different cations and anions, or by fine-tuning the alkyl chains of cations or anions, ionic liquids with different properties are formed [29]. With the deepening of research and the development of different anions and cations, the combination of existing anions and cations can reach up to 10^18^. If the traditional trial-and-error methods is still followed to find suitable ionic liquids, it will inevitably be difficult to apply due to the huge workload. How to screen suitable ionic liquid absorbents has become a challenge and difficulty [30]. At present, the methods for obtaining gas solubility data in ionic liquids are mainly divided into three categories: experimental determination, thermodynamic model prediction, and molecular simulation calculation. Among them, the experimental measurement accuracy is the highest, but the operation is cumbersome. The experimental cycle is long; the cost of reagents and consumables is high. The massive ionic liquid combination system is difficult to test one by one. The parameter fitting of thermodynamic empirical equations (such as the PR equation of state and the NRTL equation) is difficult and only applicable to specific systems, with extremely poor universality. Molecular dynamics simulation and quantum chemistry have high computational power consumption and low computational efficiency, making it difficult to meet the needs of large-scale and rapid screening. The limitations of traditional technological methods severely restrict the rapid development of new functional ionic liquids and the optimization and upgrading of gas capture processes.

In recent years, with the advancement of computer science and technology, the combination of big data and artificial intelligence has been referred to as the “fourth scientific paradigm” [31]. As a core branch of artificial intelligence [32,33,34], machine learning shows at mining implicit correlations in high-dimensional data, constructing nonlinear structure–activity relationships, and accurately fitting the inherent laws of ionic liquid structural parameters, operating conditions, and gas solubility based on massive experimental data without the need to clarify physical thermodynamic mechanisms, achieving rapid and accurate prediction of gas solubility. Compared with traditional methods, machine learning has high modeling efficiency, high prediction accuracy, and wide adaptability. It can break through the computational and experimental bottlenecks of traditional methods and provide a new technological path for high-throughput screening, absorption performance prediction, and structural orientation design of massive ionic liquids. In recent years, with the continuous improvement of chemical databases and the continuous iteration of machine learning algorithms, the application of machine learning in the field of predicting the solubility of gases in ionic liquid has become increasingly widespread, covering solubility prediction of single gas and multi-component mixed gases, as well as various algorithm systems such as conventional machine learning, deep learning, and ensemble learning, forming a complete modeling and application system. Machine learning methods have been applied in multiple directions, such as discovering new materials, predicting material and molecular properties, studying atomic force fields, and designing drugs [35,36,37,38,39,40,41,42,43]. At present, a large number of studies have focused on machine learning to predict gas solubility in ionic liquids [30,42,43,44,45,46,47,48,49], and there are also some reviews that summarize the research on some gases, such as CO_2_, H_2_S, and so on. However, there is a lack of systematic sorting of the technical system, research progress, existing problems, and development trends. Based on this, this article will systematically review the basic characteristics and advantages of gas capture in ionic liquids, the core algorithm principles of machine learning, the entire process technology system of solubility prediction, classify and summarize the research progress of machine learning prediction of various typical gases’ solubility in ionic liquids, analyze the current research problems and difficulties, and look forward to future development directions, aiming to provide comprehensive and systematic guidance and reference for subsequent related research.

## 2. Basic Principles and Workflow of Machine Learning

### 2.1. Machine Learning

Machine learning is a technology of artificial intelligence that relies on computer algorithms to mine potential patterns from massive amounts of data, construct predictive models, and achieve intelligent prediction of unknown data. This method does not require pre-set physical mechanisms and can accurately fit high-dimensional, nonlinear structure–activity relationships through data training alone. Machine learning can be mainly classified as four categories: supervised learning, semi-supervised learning, unsupervised learning, and reinforcement learning [50,51,52,53,54,55,56,57].

**Figure 1:**
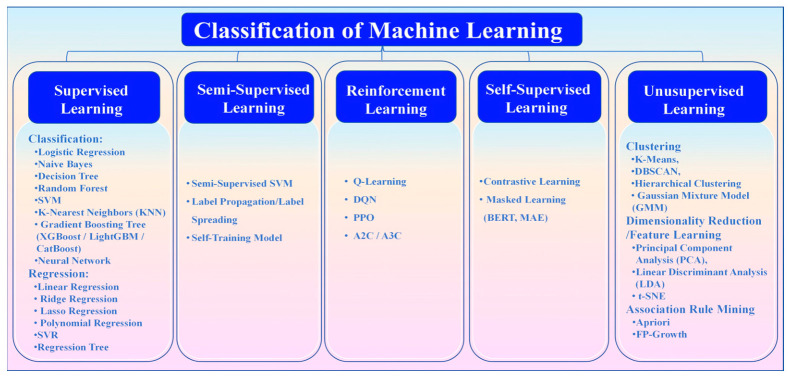
Classification of machine learning methods.

The classification is shown in Figure 1. Supervised learning trains on labeled known datasets establishes the association between features and labels and then completes classification or regression tasks on unknown data. It can be divided into regression algorithms and classification algorithms. Common algorithms include RF, DT, NN, XGBoost, SVM, EL, NB, NNB and KNN. The input data for unsupervised learning does not contain labels and relies on sample similarity to complete data partitioning. Typical algorithms include clustering algorithms, auto-encoders, GMM, and PCA. Semi-supervised learning combines the characteristics of unsupervised learning and supervised learning. The dataset consists of a large amount of unlabeled data and a small amount of labeled data, which can be used to further improve model performance. Mainstream algorithms include self-training algorithms, graph-based semi-supervised algorithms, and semi-supervised support vector machines. Reinforcement learning optimizes strategies based on reward and punishment signals by interacting with the state, actions, and rewards of the agent and the environment, in order to achieve higher returns. Representative algorithms include Q-learning and time difference learning.

### 2.2. Process of Machine Learning in Gas Capture Field

**Figure 2:**
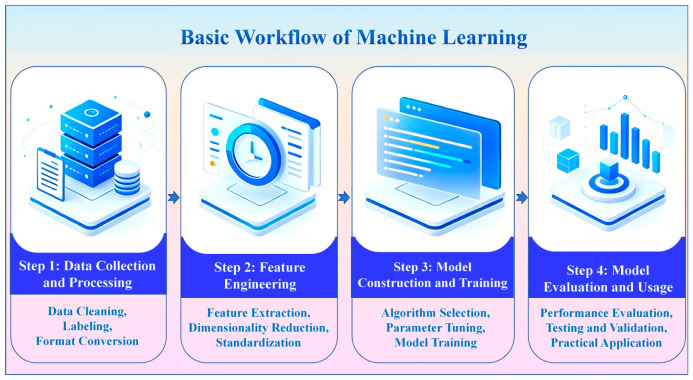
General process of machine learning modeling.

The application of machine learning in the field of gas capture has a standardized implementation process [58,59,60], which mainly includes four core steps: data collection and processing, feature engineering, model construction and training, and model evaluation and application. The overall process framework is shown in Figure 2.

**(1) Data collection and processing.** Machine learning is a typical data-driven technology whose performance is directly governed by the quality and reliability of input data. Data quality therefore serves as a core determinant of the reliability and generalization ability of machine learning research results. In machine learning modeling, datasets with insufficient samples are prone to overfitting, which severely compromises the validity and practical value of model predictions. While expanding the sample size can effectively improve model prediction accuracy, such improvements tend to saturate after a certain threshold, with no further statistically significant enhancement.

In the field of gas capture in ionic liquids, research data can be acquired from diverse credible sources. Common data repositories include authoritative standard databases such as the NIST Ionic Liquid Database, as well as peer-reviewed journal articles and academic monographs. Furthermore, natural language processing (NLP) technology [58,59] enables the automatic extraction of effective data from published literature, which substantially reduces the time and labor costs of manual data collection. Experimental testing and numerical simulation calculations also provide reliable supplementary data sources to enrich the dataset.

To guarantee high-quality dataset construction, systematic data preprocessing is essential prior to model training, which mainly includes data cleaning, standardization, and conversion. Data cleaning aims to eliminate noisy data and abnormal samples; combined with visualization tools such as box plots, it enables the accurate identification and removal of outliers. Data standardization unifies the dimensional scales of diverse feature variables, accelerates model convergence, and avoids biased weight assignment caused by feature magnitude discrepancies. Data conversion is primarily implemented for classification modeling, which standardly converts categorical variables into numerical variables to meet the unified input requirements of machine learning models.

**Figure 3:**
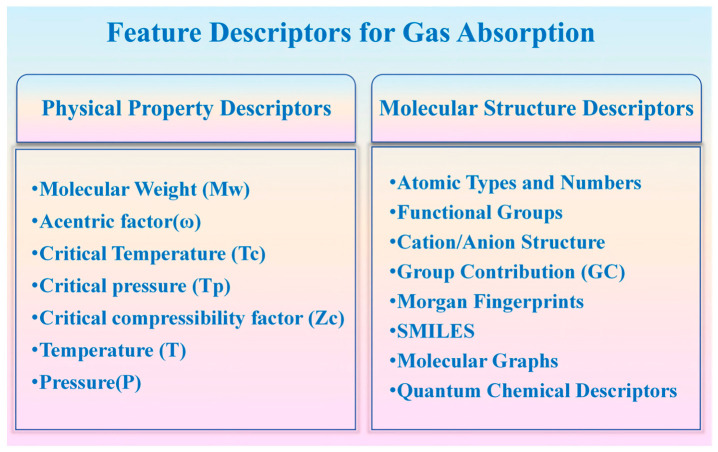
Main feature engineering for gas solubility in ionic liquids with machine learning.

**(2) Feature Engineering.** The core of feature engineering is to select measurable and quantifiable material feature descriptors and construct accurate quantitative structure–property relationship (QSPR) models [58,59,60], which is a pivotal step to ensure the prediction accuracy and generalization performance of machine learning models. Feature descriptors are core characteristic parameters extracted from research systems. For the studies of gas solubility in ionic liquids, these descriptors are primarily categorized into two types: physical property descriptors and molecular structure descriptors. Physical property descriptors cover macroscopic physical characteristics and operational parameters, including critical pressure (Pc), molecular weight (Mw), critical temperature (Tc), acentric factor (ω), critical compressibility factor (Zc), system temperature (T), and system pressure (P). Compared with physical property descriptors, QSPR models established based on molecular structure descriptors exhibit higher accuracy, better robustness, richer feature dimensions, and stronger adjustability. As a result, molecular structure descriptors have become the mainstream of current research, as illustrated in Figure 3.

The molecular structure descriptors for gas capture in ionic liquids can be further classified into eight categories:

**Atomic type and number descriptors.** All molecular substances, including ionic liquids, are composed of fundamental atoms. Accordingly, the type and quantity of constituent atoms serve as basic molecular descriptors. Typical examples include the number of carbon atoms in cations, carbon and hydrogen atoms in anions, as well as nitrogen, sulfur, oxygen, fluorine, chlorine, and boron atoms contained in ionic liquid molecules.

**Functional group descriptors.** These descriptors classify molecular characteristics based on organic molecular functional groups, with evaluation indicators covering atomic types, molecular branch lengths, heteroatom quantities, and substituent numbers, which effectively reflect the structural differences in functional groups in molecules.

**Cation and anion descriptors.** This category independently characterizes cations and anions by adopting their respective structural parameters as feature descriptors. Representative parameters include anion type, the number of specific fluorine atoms in anions, alkyl chain length of ionic groups, the quantity of specific fluorine atoms and alkyl chains in cations, and the number of ring substituents.

**Group contribution (GC) method.** This method is based on the core assumption that identical functional groups in different molecules contribute equally to macroscopic molecular properties. It possesses the advantages of simple calculation processes and excellent universal applicability in molecular property prediction.

**Molecular graph descriptors.** Molecular graphs are powerful molecular representation tools, where nodes represent atoms and edges represent chemical bonds. Graph Neural Networks (GNNs) are specialized neural networks for graph data processing, which characterize graph information through three core dimensions (nodes, edges, and global graph attributes) and adopt adjacency matrices to represent topological structures. GNN takes complete molecular graphs as both input and output and optimizes node and edge attribute vectors via the message-passing mechanism. According to differences in operational mechanisms, mainstream GNN variants are divided into graph convolutional networks (GCN), graph attention networks (GAT), and graph isomorphism networks (GIN).

**Molecular fingerprint descriptors.** Molecular fingerprints (FPs) adopt sparse vectors to characterize the presence or absence of specific substructures in molecules. As a classic feature representation method in chemoinformatics, Morgan fingerprints generate binary bit vectors by capturing local molecular environmental information. This method features fast computation speed and superior capability in retaining key molecular structural characteristics.

**SMILES descriptors.** The Simplified Molecular Input Line Entry System (SMILES) is a standardized specification that adopts ASCII strings to explicitly describe three-dimensional molecular structures. SMILES strings can be recognized and converted into two-dimensional molecular graphics or three-dimensional structural models by most mainstream molecular editing software, facilitating automated molecular feature extraction and modeling.

**Quantum chemical descriptors.** These are numerical parameters derived from molecular quantum mechanical calculations, which can comprehensively characterize molecular structural features, energy distribution, charge distribution, and chemical reactivity. Numerous studies have verified that QSPR machine learning models based on molecular structure descriptors outperform those relying on physical property descriptors in terms of accuracy and robustness. Combined with target experimental indicators (e.g., CO_2_ absorption capacity, solubility, and Henry’s constant), such models can provide effective guidance for the screening, structural design, and optimization of high-performance ionic liquids.

Feature screening is generally divided into two mainstream approaches: manual screening and algorithm-based screening. Manual screening relies on professional domain knowledge to qualitatively and quantitatively evaluate the correlation between individual features and target attributes, so as to filter invalid and low-correlation features. For algorithm-based screening, univariate feature selection methods only consider the linear correlation between a single feature and the target variable while ignoring interactive effects among features, with classic algorithms including the chi-square test, F-test, and least absolute shrinkage and selection operator (LASSO) [61]. Tree-based feature selection methods [62,63] adopt ensemble models such as random forest (RF), extreme gradient boosting (XGBoost), and light gradient boosting machine (LightGBM) to calculate feature importance and screen high-value effective features. Recursive Feature Elimination (RFE) is another efficient screening strategy that iteratively removes redundant and irrelevant features through cyclic training and verification.

**(3) Model construction and training.** When dealing with different tasks, it is often difficult to determine which model is necessarily the best choice. Therefore, for a certain task, it is usually necessary to establish multiple models and to select the final prediction model by comparing their performance [64]. In the process of model training, in order to ensure the robustness and effectiveness of the model, machine learning usually requires a large amount of data to be used as the training set and another small amount of data to be used as the test set. In addition, a validation set can be used to evaluate and adjust a small number of model hyperparameters. The training set is the underlying mechanism and rules by which a model learns features that affect the target variable from data samples provided for model learning. Sometimes, during training, a portion of the training set is further divided as a validation set to optimize model parameters and prevent overfitting. The test set is a dataset that is completely independent of the training process and is used to evaluate the generalization ability of the model, ensuring that the model performs well on a completely new dataset [65]. There are many machine learning algorithms used in model construction to build gas solubility models. Detailed algorithms and their advantages and disadvantages are provided in Table S1 in Supplemental Materials.

**(4) Model Evaluation and Use.** Evaluating the performance of developed machine learning models is crucial. Equally important is to choose appropriate indicators to help explain the model’s predictions. The evaluation of model performance usually relies on a series of evaluation indicators. Some commonly used evaluation metrics in classification and regression models [61,62,63,64] include F1 score, recall, accuracy, ROC curve, AUC value, as well as R^2^, MSE, RMSE, MAPE, ARD, AAD, STD, and AARD%, which are also used to evaluate the performance of prediction models. Different evaluation indicators can help researchers evaluate the performance of models from different perspectives, selecting different indicators to evaluate models based on specific research questions, data attributes, and model objectives. But usually, a good model evaluation will combine multiple indicators to comprehensively understand the performance of the model, as relying on a single indicator may be misleading. Some models may perform well on one metric but perform poorly when evaluated using other metrics. The model with the best comprehensive evaluation effect will be saved as the final prediction model. There are no strict rules for selecting indicators, but authors should prove that their choices are reasonable. All prediction results should be validated by experimental validation to take advantage of machine learning technology.

## 3. Gas Solubility in Ionic Liquids Studied by Machine Learning

Ionic liquids have the advantages of a wide liquid range, extremely low volatility, strong chemical stability, outstanding gas solubility, designable structure, easy recovery, and environmental friendliness. They have a great potential for application in greenhouse-gas treatment fields such as carbon dioxide capture and storage, industrial waste gas purification, and gas separation and recovery. With the increasing requirements for environmental protection and energy efficiency, research on the use of ionic liquids for gas capture has rapidly developed, and good results have been achieved in the treatment of acidic gases such as CO_2_, NH_3_, and H_2_S and other gases [66,67,68,69,70,71,72,73,74,75,76,77,78,79,80,81,82,83,84,85,86,87,88,89,90,91,92,93,94,95,96,97,98,99,100,101,102,103,104,105,106,107,108,109,110,111,112,113,114,115,116,117,118,119,120,121,122,123,124,125,126,127,128,129,130,131,132,133,134,135,136,137,138,139,140,141,142,143,144,145,146,147,148,149,150,151,152,153,154,155,156,157,158,159,160,161,162,163,164,165,166,167,168,169,170,171,172,173,174,175,176,177,178,179,180,181,182,183,184,185,186,187,188,189]. However, the combination of ionic liquid structures is complex, and traditional experimental methods are difficult to clarify the relationship between microstructure and macroscopic properties. Conventional QSAR models are often constructed based on simplified assumptions, and gas solubility is influenced by multiple factors such as ion structure, temperature and pressure, and inter-molecular interactions. These factors exhibit complex nonlinear relationships and are difficult to accurately characterize using traditional models. At the same time, this type of model is limited by experimental data and has weak generalization performance. Machine learning has powerful nonlinear fitting, data processing, and generalization capabilities, providing new ideas for such problems. Currently, it has been widely applied in research on the solubility of various gases in ionic liquids and has made progress. The following text will summarize and explore the current research status in this field.

### 3.1. CO_2_ Solubility

While the global economy continues to develop, resource scarcity and environmental pressures are becoming increasingly prominent, and optimizing the energy structure has become the core direction to solve current problems. Carbon dioxide is the main greenhouse gas, and excessive emissions can exacerbate the greenhouse effect. At the same time, it is also an important one carbon (C1) resource that can be applied to produce high value-added chemicals and is widely applied in many fields such as oil field displacement, refrigeration, welding and casting, and firefighting. Therefore, the capture, conversion, and resource utilization of carbon dioxide have become hot topics in the global scientific research field. Ionic liquids have become an ideal medium for absorbing CO_2_ due to their low melting point, excellent physical and chemical stability, and strong gas solubility. Currently, relevant research has achieved fruitful results. Yang et al. [66] published the first review on the scientific understanding of CO_2_ solubility in ionic liquids and in situ catalysis. After that, many reviews were focused on these fields [14,15,16,17,18]. However, the category of ionic liquids is very large, and the combination of anions and cations is complex and diverse. Traditional methods are difficult to quickly screen for systems that combine high CO_2_ solubility and conversion performance. In response to this issue, machine learning, with its powerful data mining and analysis capabilities, has been used to construct a quantitative structure–activity relationship (QSAR) between the structure of ionic liquids and their CO_2_ solubility, providing theoretical support for the screening and design of ionic liquids in CO_2_ solubility and conversion systems. This direction has made many research advances, and some reviews have been discussed.

**Figure 4:**
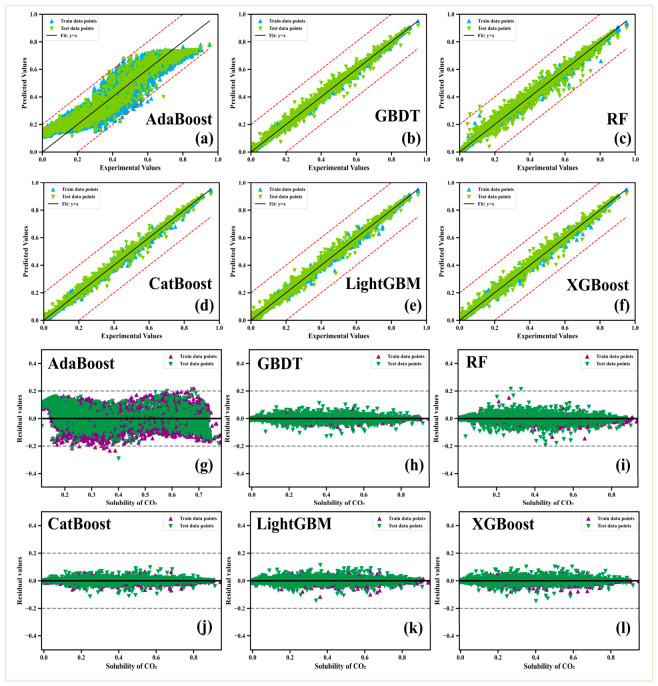
Comparison of the predicted and experimental CO_2_ solubility values in ionic liquids by different machine learning models based on FSD (**a**–**f**) and the residuals for various ensemble machine learning models (**g**,**h**). (**a**,**g**) AdaBoost; (**b**,**h**) GBDT; (**c**,**i**) RF; (**d**,**j**) CatBoost; (**e**,**k**) LightGBM; (**f**,**l**) XGBoost. First publication of the work by the Royal Society of Chemistry [71], DOI: 10.1039/D5CP01972A.

ANN is the earliest machine learning algorithm used to predict the solubility of CO_2_ in ionic liquids [67,68,69,70,71,72,73,74,75,76,77,78,79,80]. In 2011, Eslamimanesh et al. [67] applied the ANN algorithms to predict the solubility of supercritical CO_2_ in 24 common ionic liquids. They constructed an optimized three-layer feed-forward neural network model with the critical characteristics of ionic liquids, system temperature, and pressure as input parameters, and conducted modeling analysis based on 1128 sets of experimental data corresponding to 24 types of ionic liquids. The results indicate that the determination coefficient R^2^ of the model for predicting solubility can reach 0.993, with an average absolute deviation of only 3.6% from the experimental value, demonstrating excellent predictive performance. Subsequently, various machine learning algorithms were gradually applied to predict the solubility of CO_2_ in ionic liquids. Baghban et al. [68] compiled 728 sets of experimental data, using parameters such as temperature, pressure, critical pressure, critical temperature, and eccentricity factor as descriptors, and constructed prediction models for the CO_2_ solubility in ionic liquid based on ANFIS and MLP algorithms. Meanwhile, the study compared machine learning models with classical thermodynamic models such as SRK and PR, and the results confirmed that the MLP model had better prediction accuracy, with an R^2^ of 0.972 and a MSE as low as 0.000133. Shaukat et al. [69] used moisture content, pressure, temperature, and other characteristic parameters as model inputs, and CO_2_ solubility in ionic liquids as output variables. They built a prediction model based on SVM, GPR, and other algorithms. The results indicate that the model constructed by the GPR algorithm has the best prediction accuracy, while the SVM model has the worst comprehensive prediction performance. Liu et al. [70] summarized 1517 sets of CO_2_ solubility experimental data of 20 ionic liquids, using temperature, pressure, critical temperature, and other physical parameters as input features. They combined GWO, PSO, and SSA to optimize SVR models and constructed three different mixed prediction models. Research verification shows that the PSO-SVR model has the best predictive performance among all models. For the practical application scenarios of ionic liquids, we constructed FSD and dimensionless descriptors of CORE based on the GC methodand explored the feasibility of model dimensionality reduction [71], the prediction performance and residual distribution of each model are shown in Figure 4. 

In our work [71], six ensemble learning models were used to predict the CO_2_ solubility in ionic liquid, and the results showed that CatBoost had the best comprehensive performance. The comparison is valid only within the original study, dataset, descriptor set, and validation protocol. Its models based on FSD and CORE descriptors had R^2^ values of 0.9945 and 0.9925, respectively, and MAE values of 0.0108 and 0.0120, respectively. The key features of the CatBoost CORE model were identified through interpretability analysis. These works indicate that machine learning has achieved good results and effectiveness in the construction of CO_2_ solubility prediction models in ionic liquids. Based on the CORE descriptor, the best experimental conditions are obtained, and nine kinds of ionic liquids with superior performance are recommended.

More relevant research progress [67,68,69,70,71,72,73,74,75,76,77,78,79,80,81,82,83,84,85,86,87,88,89,90,91,92,93,94,95,96,97,98,99,100,101,102,103,104,105,106,107,108,109,110,111,112,113,114,115,116,117,118,119,120,121,122,123,124,125,126,127,128,129,130,131,132,133,134,135,136,137,138,139,140,141] is summarized in Table 1 and Table S2. According to the table, existing models often use temperature, pressure, eccentricity factor, component concentration, mass fraction, chemical structure, and ionic liquid cation and anion configurations as input descriptors. The mainstream algorithms include SVR, MLP, LSSVM, CART, BPNN, etc. Among them, LSSVM and CART models have particularly outstanding prediction accuracy. The model constructed based on CART and LSSVM has a coefficient of determination approaching 1, and the prediction error can be basically ignored [72,73]. The MLP-ANN model also performs well, with an R^2^ of 0.9972 and an MSE of 0.000133 [68]. Combining the radial basis function and GC model, the AARD% and R^2^ reached 2.68 and 0.996, respectively [73]. The comparison is valid only within the original study, dataset, descriptor set, and validation protocol.

Overall, machine learning algorithms have excellent predictive ability for CO_2_ solubility in ionic liquids. There are significant differences in the size of the datasets used in each study, with sample sizes ranging from two-digit small samples to over 18,000 large samples. Large samples can provide more comprehensive data information and make it easier to train more stable models. The dataset partitioning is mainly based on a training set: test set as 8:2, while there are also partitioning methods such as 1:1, 6:1, 9:1, etc. The partitioning ratio will directly affect the prediction accuracy. When modeling, it is necessary to optimize and debug based on the algorithm type and data volume. In terms of feature engineering, the vast majority of studies select the eight types of variables mentioned earlier as input parameters. More than 90% of studies use thermodynamic descriptors such as temperature and pressure as core inputs, a feature that was more evident in early research. Only a small number of studies have introduced ionic liquid molecular descriptors and structural parameters, including GC, SMILES, molecular graphs, fingerprints, molecular structure descriptors, and atomic, group, and structural fragments of anions and cations. Combining molecular structure descriptors with temperature and pressure parameters can achieve ideal prediction results, but increasing the number of input parameters will increase the dimensionality and complexity of the model. There are two main types of existing research: one uses multi feature input and relies on rich information to improve prediction accuracy, but requires higher computing power and runtime; Another type uses SHAP and other methods to screen core features, streamline input parameters, reduce model complexity, and ensure both performance and computational efficiency. The resulting correlation model also has stronger universal adaptability. There are various types of machine learning algorithms currently used for solubility prediction, including MLP, SVR, LSSVM, GA, CART, RF, ANN, DNN, CNN, etc. Different algorithms have their own advantages and disadvantages in performance, and there is no universal optimal algorithm. It is necessary to select based on practical problems and data characteristics. Among them, MLP, LSSVM, RF and other algorithms have achieved high prediction accuracy on multiple datasets, with R^2^ generally approaching 1 and outstanding generalization ability. For big datasets and high-dimensional feature scenes, complex models such as DNN and CNN are often used. In small samples and few-feature scenarios, simple models such as MLP and RF can achieve good results. From this, it can be seen that model selection needs comprehensive consideration of problem complexity, data characteristics, and computational resources. In addition, over 90% of machine learning models belong to black box models, which are different from traditional QSAR methods in that they have clear mathematical expressions, and most studies have not publicly disclosed algorithm codes and models, which is not conducive to the reuse and promotion of methods. Overall, existing models generally have high prediction accuracy on the test set, with R^2^ mostly reaching 0.99 or above, which can accurately predict CO_2_ solubility.

### 3.2. H_2_S Solubility

As a highly toxic industrial waste gas with a pungent odor, hydrogen sulfide (H_2_S) is widely produced in industries such as metallurgy, petrochemicals, rubber, and leather making. Capturing and treating it for resource utilization in the production of sulfuric acid, sulfur, and other products is of great practical significance. Ionic liquids, as green solvents, have become excellent media for H_2_S absorption due to their low vapor pressure, good thermal stability, and strong solubility. Therefore, the academic community has conducted extensive research on the quantitative structure–activity relationship between the structure of ionic liquids and H_2_S solubility, in order to guide the design and screening of ionic liquids. Machine learning has also gradually been applied in this field and has achieved certain results [142,143,144,145,146,147,148,149,150,151,152,153,154,155,156,157,158].

The application of machine learning to explore the solubility characteristics of ionic liquids in H_2_S has been gradually carried out since 2014. Shafiei et al. [142] selected pressure, eccentricity factor, and temperature as feature descriptors and introduced particle swarm optimization neural network and back propagation neural network algorithms to model and analyze the absorption behavior of H_2_S in ionic liquids. The dataset contains 465 samples, divided into training and testing sets in a ratio of 372:93. The test results confirm that the PSO-ANN model has higher fitting accuracy, with R^2^ and MSE of 0.99218 and 0.00025, respectively, while the BP-ANN model has two indicators of 0.95151 and 0.00335, respectively. Subsequently, Soleimani et al. [143] used this dataset, with the training set of 80% samples and the testing set of 20% samples. They established a quantitative relationship between thermodynamic descriptors and H_2_S solubility using the SGB algorithm. The final model achieved an R^2^ of 0.999543 and an MRAE of 0.022198, with extremely low prediction error. 

**Figure 5:**
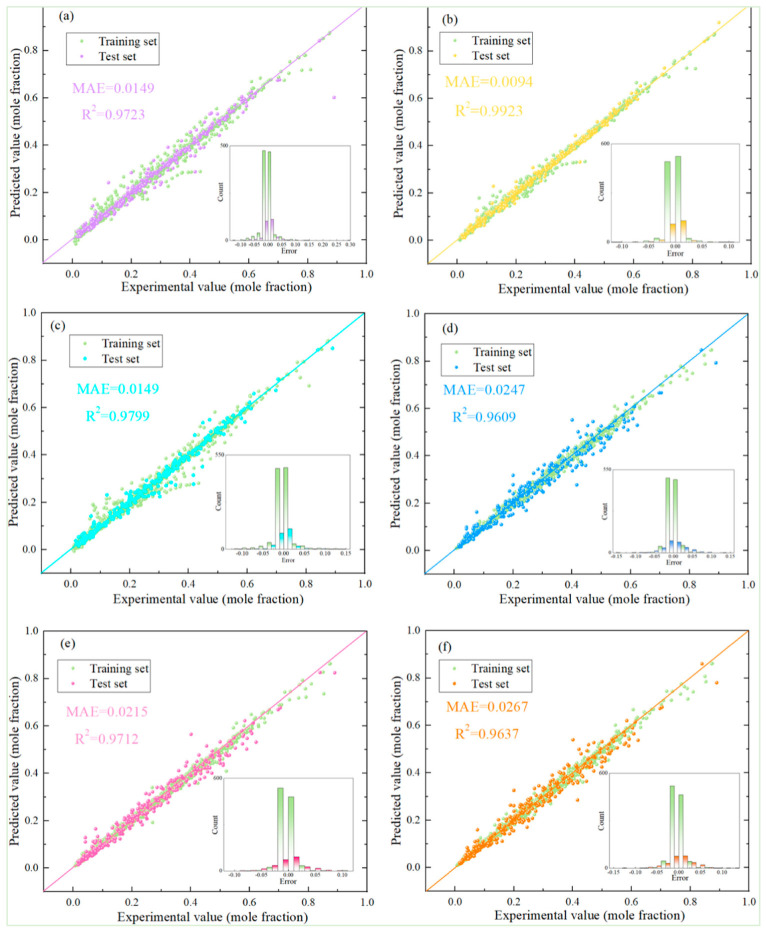
The scatter plot and error distribution histogram of the predicted and experimental value for H_2_S (**a**) DNN-MD, (**b**) DNN-MI, (**c**) DNN-MD_MI, (**d**) RF-MD, (**e**) RF-MI, and (**f**) RF-MD_MI. First publication of the work by the American Chemical Society [144], DOI: 10.1021/acssuschemeng.2c07541.

In 2023, Liu et al. [144] combined molecular feature extraction techniques to construct the Molecular Feature Identifier (MFI). Molecular descriptors (MD), MFI, and their combined features were used as structural characterization methods, and temperature and pressure parameters were input into DNN and RF models. The 465 data points were used for solubility prediction. The results indicate that MFI has the best model fit for structural characterization, followed by MD-MFI, and MD has the weakest effect. The overall prediction accuracy of DNN at the model level is higher than that of RF, among which the DNN-MFI model has the best comprehensive performance, with test set R^2^ = 0.9923, MAE = 0.0094 and RMSE = 0.0151. Figure 5 compares the results of different machine learning algorithms. In summary, multiple studies have fully validated the strong practical value and application potential of machine learning technology in the study of H_2_S dissolution in ionic liquids. It should be clearly noted that the comparison is valid only within the original study, dataset, descriptor set, and validation protocol.

More research on machine learning for predicting the solubility of H_2_S in ionic liquids is summarized in Table 2. The data in Table 2 shows that the sample size of the relevant datasets ranges from 465 to 516. The mainstream division method is to allocate the training and testing sets in an 8:2 ratio, with some studies adding additional validation sets. Similar to the research on predicting CO_2_ solubility, early models often selected physical and chemical parameters such as critical temperature, critical pressure, pressure, temperature, eccentricity factor, as well as molecular structure and component information as input features. These parameters are the core basis for predicting physical properties. Researchers have used various machine learning algorithms such as SGB, ELM, MLP, ANN, LSSVM, CNN, RNN, DBN, GP, and XGBoost to improve prediction accuracy and efficiency. Many models achieved very high prediction accuracy on the test set (such as R^2^ values close to or exceeding 0.99), indicating that these models can accurately predict target properties. The R^2^ value of the model constructed by the SGB algorithm is 0.999543, and the MRAE value is 0.022198 [145]. The model constructed by the ELM algorithm also achieved excellent results, with an RMSE of 0.0112 and an R^2^ of 0.999 [146]. The model constructed by the MLP-ANN algorithm [147] had AARD% and R^2^ of 2.3292 and 0.9982, respectively. From this, it can be seen that the H_2_S solubility model of IL constructed using the ML algorithm has good predictive performance. Overall, deep learning algorithms (such as CNN, RNN, DBN) and ensemble learning algorithms (such as SGB, XGBoost) have shown good predictive performance on multiple datasets. This indicates that these algorithms may have stronger modeling capabilities and better predictive performance when dealing with complex data and solving complex problems. It should be noted that the comparison is valid only within the original study, dataset, descriptor set, and validation protocol.

### 3.3. NH_3_ Solubility

Ammonia (NH_3_) is an important basic chemical raw material and also a polluting gas that poses a threat to human health, life, and the living environment. Therefore, the recycling and utilization of NH_3_-containing tail gas are of great significance. Green solvents, including deep eutectic solvents and ionic liquids, are widely used in NH_3_ capture research due to their advantages such as high thermal and chemical stability, low saturation vapor pressure, and designable structure and function [159,160,161]. In order to seek renewable absorption solvents with large absorption capacity and excellent absorption performance, machine learning methods have been used to establish quantitative structure–activity relationship models between the structural characteristics, experimental conditions, and absorption capacity or solubility of ionic liquids, in order to provide support and assistance for related research, which has made certain progress.

Baghban et al. [162] constructed an ML model for the prediction of NH_3_ solubility based on thermodynamic properties (TQ) such as Mw and T, etc. Liu et al. [163] analyzed the structure–activity relationship between ionic liquid functional groups and NH_3_ solubility using the micro GC. Li et al. [164] achieved an accurate prediction of the solubility of NH_3_ by calculating the σ-profiles (surface charge distribution) of ion fragments based on quantum chemistry (QC). 

**Figure 6:**
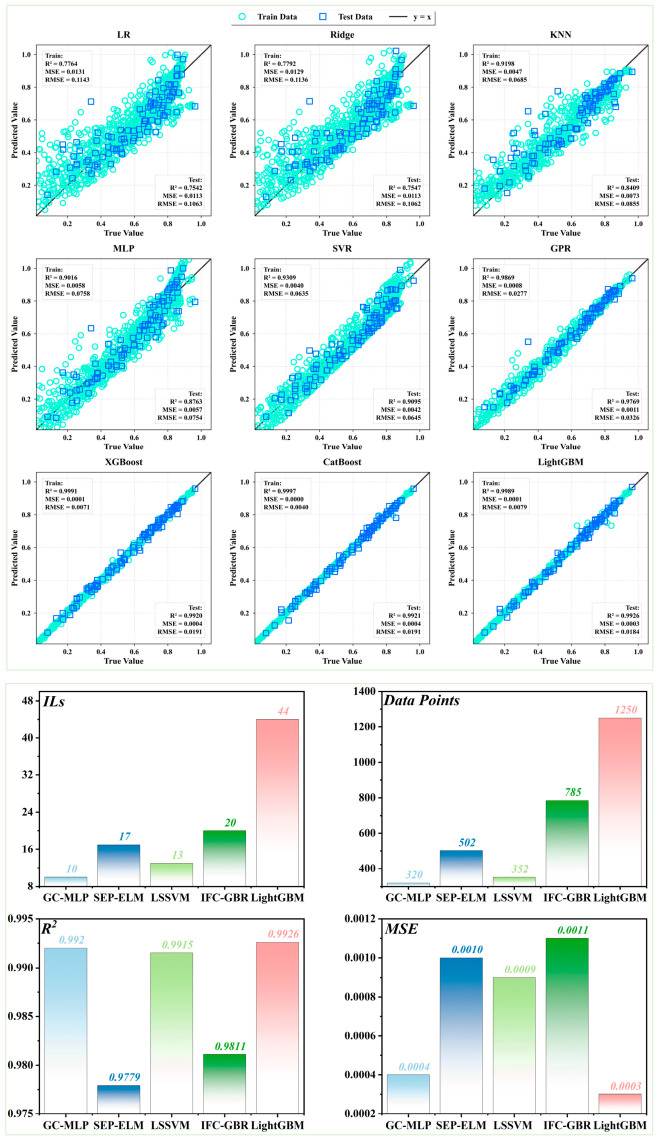
The behavior of different models studied and the comparison between the best model and other reported results. Reproduced with permission from Ref. [165] published by Elsevier.

We provided a machine learning model [165] based on SMILES molecular descriptors to attain a good prediction of the solubility of NH_3_ in ionic liquids. We built a database with 934 data points of NH_3_ experimental solubility in ionic liquids and systematically evaluated the performance of 9 machine learning models (LASSO, LR, MLP, GPR, KNN, LightGBM, SVR, XGBoost, GPR, and CatBoost) on the solubility of NH_3_ in ionic liquids. The results showed that traditional linear models (LASSO and LR) performed poorly, while ensemble learning methods demonstrated significant advantages, with the CatBoost model having the highest prediction accuracy (R^2^ = 0.9928, MSE = 0.004). Figure 6 shows the behavior of different models and the comparison between the best model and other reported results. Based on SHAP analysis, the pressure (P/Bar), temperature (T/K), atomic charge surface area distribution (PEOE_VSA9), the number of aromatic rings (NumAromaticRings), molar refractive index surface area distribution (SMR_VSA4), number of free radical electrons (NumRadicalElectrons), molecular lipid-water partition coefficient (MolLogP) and van der Waals surface area descriptor based on E-State index (VSA_EState3) are key factors primarily influence NH_3_ solubility in ionic liquids. Based on the key factors and the best model, a total of 1773 novel ionic liquids were designed and screened.

More research progress on machine learning for forecasting the ammonia solubility in ionic liquids is summarized in Table 3. From Table 3, the sample size of relevant studies ranges from 320 to 1356 groups, and the dataset is generally divided into an 8:2 ratio. The input features of the model are mainly divided into two categories: one is thermodynamic parameters such as pressure, temperature, critical parameters, molecular weight, eccentricity factor, etc. Another type is chemical structure-related features, including molar concentration, hydrogen bond donor/acceptor type, water content, component mass fraction, charge surface area, etc. The existing machine learning algorithms used in research mainly include ELM, MLP, CFFNN, and ANFIS. In 2020, Kang et al. [166] used electrostatic potential surface area, temperature, and pressure as inputs to conduct modeling and prediction using ELM. The model’s coefficient of determination R^2^ reached 0.9937, and the AARD% was only 2.95%, confirming the excellent performance of ELM in this type of prediction task. In 2022, Sun et al. [167] constructed a model based on CFFNN and obtained an R^2^ of 0.995 and an AARD% of 4.98%. Multiple studies [163,168] have also confirmed the excellent predictive ability of MLP: its training set mean absolute error (MAE) is 0.0004 and R^2^ is 0.992. In another study, the MLP model training set had an R^2^ of 0.9967 and an AARD% of 1.23697. The above results demonstrate that MLP combines flexibility and high accuracy in parsing complex data and predicting target properties. In addition, RF, SVM, PSO-ANFIS, DNN, GRNN, WNN, RBF and other algorithms have also been applied in this field and achieved good results. It should be noted that the comparison is valid only within the original study, dataset, descriptor set, and validation protocol.

Overall comparison shows that ELM and MLP have advantages in overall stability and prediction accuracy under different experimental conditions. From the perspective of model complexity, although deep learning models such as DNN have stronger theoretical modeling capabilities, relatively simple models such as ELM and MLP can also achieve high-precision prediction for small and medium-sized sample datasets, such as ammonia solubility. This indicates that model selection needs to be comprehensively considered based on factors such as the complexity of the research problem, data characteristics, and computational resources. Overall, the ammonia solubility model in ionic liquids constructed based on machine learning generally has good predictive performance.

### 3.4. Solubility of Other Gases

Sulfur dioxide and nitrous oxide are common intermediate products in industrial production and chemical experiments, and their emission concentrations directly affect the atmospheric environment and climate conditions. Hydrogen, methane, and oxygen are the main components of clean energy, natural gas, and the basic substances that sustain life activities, respectively. Therefore, developing green and efficient absorption and separation technologies for the above-mentioned gases is of great practical significance. Ionic liquids, as a new type of green and efficient solvent, have strong solubility, high structural designability, low vapor pressure, and excellent thermal stability. They not only exhibit excellent solubility for hydrogen sulfide, ammonia, and carbon dioxide, but also have good solubility and separation selectivity for various single gas and mixed systems. However, ionic liquids have a wide variety of combinations of anions and cations, and a wide range of categories. It is difficult to select high solubility systems that are suitable for specific gases from them. At present, machine learning has successfully constructed a quantitative model between the structural characteristics of ionic liquids, experimental conditions, and the solubility/adsorption properties of hydrogen sulfide, ammonia, and carbon dioxide, providing an effective approach for targeted screening of functional ionic liquids. In recent years, this method has gradually expanded its application to research on gases such as sulfur dioxide, nitrous oxide, hydrogen, methane, and oxygen.

Bahmani et al. [170] used ANN to construct a model for predicting the sulfur dioxide solubility in different ionic liquids. They selected pressure, temperature, critical pressure, critical temperature, and eccentricity factor as input variables to establish a correlation model between each parameter and SO_2_ solubility. The test results show that the ANN model has excellent prediction performance, with a mean square error (MSE) as low as 0.0004 and a determination coefficient R^2^ of 0.9861. Based on the group contribution (GC) descriptor, Baghban et al. [171] combined LSSVM to optimize the SO_2_ solubility prediction model. The model takes pressure, temperature, and the structural characteristics of 17 ionic liquids as inputs, with a focus on the effect of molecular structure on the performance of SO_2_ solubility. They found that the LSSVM model has excellent predictive behavior, with an R^2^ of 0.995 for the test set and an MSE approaching zero. Mokarizadeh et al. [172] built prediction models based on LSSVM and ANN, with input variables including temperature, pressure, boiling point, critical temperature, critical compression factor, critical pressure, eccentricity factor, and a series of thermodynamic parameters. By comparison, LSSVM has a more prominent predictive effect on SO_2_ solubility, with an R^2^ of 0.986 for the test set and an RMSE of 0.025. It should be noted that the comparison is valid only within the original study, dataset, descriptor set, and validation protocol.

**Figure 7:**
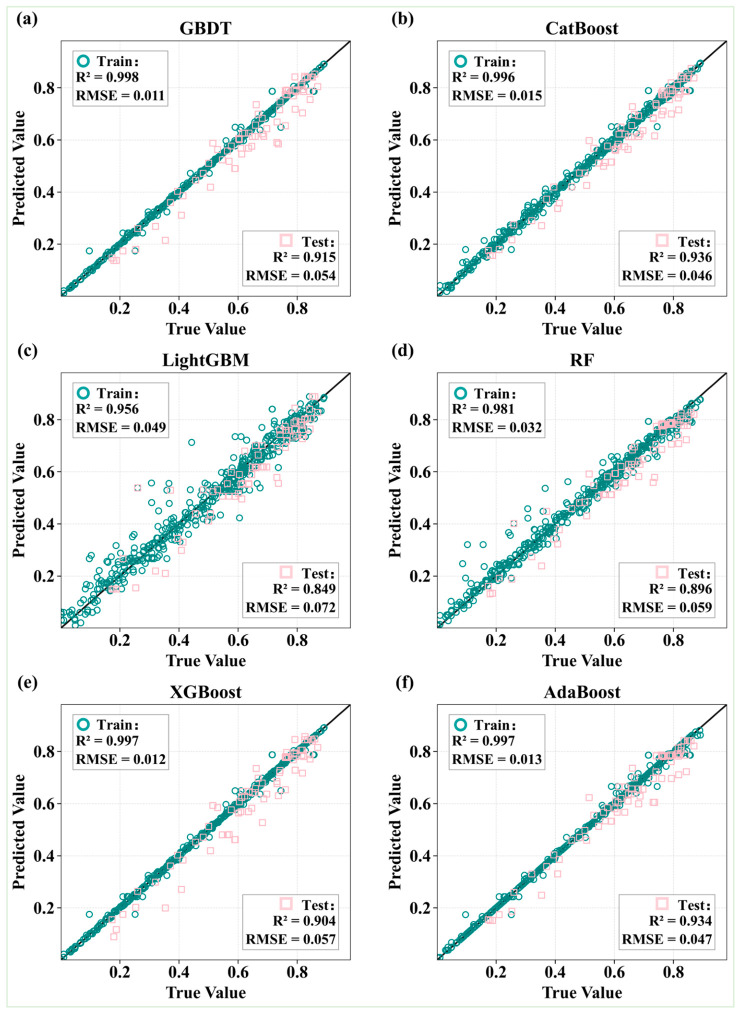
Comparison between the predicted values and the experimental values of the different models for SO_2_ in ionic liquids. (**a**–**f**) respectively refer to the CatBoost, LightGBM, XGBoost, GBDT, RF and AdaBoost models.

Zhao et al. [173] used CNN and RF algorithms to establish predictive models for temperature, pressure, ionic liquid structural parameters, and SO_2_ solubility. The results in Figure 7 show that CNN has the best comprehensive performance, with a test set R^2^ of 0.983, while balancing prediction accuracy and model interpretability; The RF model performs slightly weaker, with a test set R^2^ of 0.970. We have independently constructed a brand new SO_2_ absorption dataset, covering 166 ionic liquids and a total of 564 sets of SO_2_ absorption capacity data. Based on the structure descriptors of anions and cations, a machine learning prediction model was established using six algorithms: RF, AdaBoost, CatBoost, GBDT, XGBoost, LightGBM, and feature importance analysis was conducted. It showed that all six models had good predictive ability, among which XGBoost had the best performance, with an R^2^ of 0.962 for the test set and the MAE of 0.013. Figure 4 shows the scatter comparison between the predicted values and the experimental values of the six models. The comparison is valid only within the original study, dataset, descriptor set, and validation protocol.

Table 4 summarizes relevant literature on machine learning for predicting the solubility of gases such as nitrous oxide, sulfur dioxide, hydrogen, oxygen, and alkanes in ionic liquids. Based on the research information in the comprehensive table, the following can be summarized. For the prediction of N_2_O solubility, the model inputs mainly include thermodynamic properties such as temperature, pressure, and critical parameters. The sample size of the existing datasets used for modeling gas solubility ranges from 25 to 743 groups, and the overall sample size is relatively small, reflecting the limited experimental research in this field. Small sample datasets can easily constrain the generalization ability of models, while sufficient data is more conducive to training prediction models with stronger stability and wider adaptability. The input features selected for each study include parameters such as pressure, temperature, critical temperature and critical pressure of ionic liquids, eccentricity factor, critical compression factor, molecular weight, moisture content, and molecular structure. Feature screening is the key to improving model prediction accuracy, and many studies have improved model performance by optimizing input feature combinations. For example, in H_2_ and O_2_ solubility prediction, the fusion of molecular structure and thermodynamic dual features has ultimately achieved ideal prediction results.

The commonly used algorithms in this field include artificial neural networks (ANN), cascaded feed-forward neural networks (CFNN), feed-forward neural networks (FFNN), etc. [173,174,175,176], among which the CFNN model performs the best, with a determination coefficient R^2^ of 0.999 or above [173]. In the prediction of SO_2_ solubility and modeling of gas separation, least squares support vector machine (LSSVM), recurrent neural network (RNN), and deep belief network (DBN) have also been applied. Comparison shows that LSSVM has the best comprehensive performance, with a model R^2^ of 0.995 [174]. The comparison is valid only within the original study, dataset, descriptor set, and validation protocol. Overall, research on the solubility characteristics of gases such as SO_2_, N_2_O, and alkanes in ionic liquids is still relatively scarce, and further in-depth and systematic exploration is needed.

## 4. Discussion

The previous section systematically reviewed the application of machine learning methods in the field of ionic liquid absorption of various gases. Based on the integration and sorting of existing relevant research results, this article summarizes five dimensions: model evolution, dataset, feature engineering selection, model algorithm and parameter optimization, and model performance evaluation.

(1). Characteristic equation construction. Table 1, Table 2, Table 3 and Table 4 and Table S2 reveal an iterative upgrading trend of models adopted for the prediction of gas solubility in ionic liquids. Early studies mainly employed the LR-QSAR model for performance prediction. With the development of machine learning, nonlinear algorithms including XGB, ANN, RF and SVM have been widely applied to address the accuracy defects of traditional linear models and adapt to high-dimensional complex data, constructing robust nonlinear regression prediction frameworks. To further improve prediction accuracy, advanced deep learning methods such as RNNs and GNNs have been increasingly utilized. These models can automatically extract effective latent features from raw data and integrate them for modeling, greatly enhancing the prediction accuracy of gas capture in ionic liquid performance, and providing reliable technical support for ionic liquid performance prediction and structural optimization.

(2). Data and quality. Data quality is a decisive factor for machine learning research. As newly developed green solvents, ionic liquids have a short research history in gas capture, and their experimental datasets suffer from inherent uncertainties, cross-source inconsistency, category imbalance, sparse data in specific temperature and pressure ranges, and potential data leakage. These flaws restrict the prediction reliability and engineering application value of relevant machine learning models. First, experimental uncertainties generate unquantifiable data noise. Operational fluctuations, instrument errors, manual deviations and inherent ionic liquid properties lead to inconsistent replicate data, while most public datasets lack uncertainty annotation, further deteriorating data quality and model accuracy. Second, datasets from different literature and databases adopt inconsistent experimental protocols, calibration and preprocessing standards. Differences in test conditions and sample pretreatment cause severe data heterogeneity, hindering the development of universal and robust prediction models. Third, existing datasets present an obvious imbalance and long-tail distribution. Data are mostly concentrated on conventional ionic liquid systems and CO_2_ absorption, while data for novel functionalized ionic liquids and special gas capture (SO_2_, NH_3_, hydrocarbons) are extremely scarce, with few samples for minority systems and extreme operating conditions. This distribution bias limits model learning of niche system characteristics and extreme condition variation rules, weakening model generalization. Finally, incomplete feature dimensions, absent unified industrial data standards and non-standardized modeling preprocessing easily trigger data leakage, resulting in overestimated model performance and poor practical generalization, which undermines the credibility of machine learning in ionic liquid design and absorption prediction. Overall, high-quality, standardized annotated datasets are still insufficient, and solving data bottlenecks will be a key research focus in the future.

(3). Input features. Diverse input features are currently used for the prediction of gas solubility in ionic liquids. As molecular structures fundamentally determine substance properties, integrating solubility-correlated physicochemical and structural descriptors is the core of accurate solubility prediction modeling. However, excessive features increase model dimensionality and computational complexity. To solve this issue, studies adopt SHAP-based feature screening to select core parameters and optimize feature combinations, which simplifies model structure and improves prediction performance, guiding the screening and design of new gas absorbents. For example, combining molecular structures and thermodynamic characteristics can achieve excellent prediction results for H_2_ and O_2_ solubility, verifying that rational feature screening and combination optimization are vital to improving the overall performance of solubility prediction models.

(4). Machine learning algorithms. Algorithm selection and optimization. Multiple machine learning algorithms including SVM, FFNN, LSSVM, CFNN, DBN, KNN, GBR, RF, ELM, RNN, MLP, CatBoost and XGBoost have been applied for solubility prediction. Each algorithm has unique strengths and limitations in applicable scenarios and prediction performance (Table S1), and no single algorithm can fit all research cases. Thus, researchers need to flexibly select algorithms based on data characteristics and research complexity. SVM performs well in small-sample modeling, while CFNN is superior in fitting complex nonlinear relationships between ionic liquid structures and gas solubility. Hyperparameter optimization via cross-validation, grid search and Bayesian optimization is critical to mitigating overfitting and underfitting and improving model generalization. In addition, model ensemble strategies integrate the complementary advantages of multiple base models through weighting and voting, further boosting prediction accuracy and stability. However, existing models generally have poor extrapolation ability. When ionic liquid structures, gas types or operating conditions exceed the training data range, prediction errors rise sharply, failing to conform to physical laws and producing large deviations. Improving model extrapolation performance is therefore a key challenging research direction for future studies.

(5). Model performance evaluation. Model performance evaluation metrics. Scientific evaluation is essential for machine learning model development, and reasonable metric selection is the premise of objective performance assessment. Common metrics are divided into scale-dependent and scale-independent types. Precision, recall and F1-score apply to classification models, while R^2^, MSE, RMSE and AARD% are mainstream criteria for regression-based gas solubility prediction.

There are no unified metric selection standards in current academic research, yet studies need to clarify the rationality of their evaluation systems. R^2^, AARD% and RMSE are the most widely used metrics for the prediction of gas solubility in ionic liquid, supplemented by MAE, standard deviation, ARD% and MAPE for multi-dimensional quantitative evaluation. Nevertheless, existing studies rarely elaborate on metric selection rationale, and unified quantitative error thresholds are not well defined. Multi-metric joint evaluation is widely adopted to avoid the one-sidedness of single-metric assessment, eliminate evaluation bias, and ensure the comprehensiveness, objectivity and credibility of model performance evaluation for solubility prediction.

## 5. Conclusions and Outlook

This paper systematically reviews the applications and development of machine learning techniques for gas solubility prediction in ionic liquids, focusing on feature screening strategies, mainstream modeling algorithms, and model predictive performance. Machine learning input features in this field fall into four core types: thermodynamic parameters (temperature, pressure, component mass fraction, etc.), microstructural properties of anions and cations (functional groups, atomic number information, etc.) of the ionic liquids, molecular fingerprints and spectral features, and quantum chemical descriptors (molecular polarity, charge distribution, energy characteristics, etc.). Existing studies adopt single or combined multi-type features to build machine learning-based gas solubility prediction frameworks.

In terms of modeling algorithms and performance, ensemble learning and deep learning show excellent applicability for the prediction of the gas solubility in ionic liquids. Gradient boosting-based ensemble methods (LightGBM, XGBoost, random forest) deliver high prediction accuracy and robustness. Classic deep learning architectures, including ordinary neural networks, graph neural networks, convolutional neural networks and graph isomorphism networks, effectively capture the complex nonlinear correlations between ionic liquids properties and gas solubility. Overall, machine learning has become a core tool for accurate prediction of gas capture performance in ionic liquids, offering a new paradigm for data mining, intelligent modeling and quantitative analysis in this research area.

**Figure 8:**
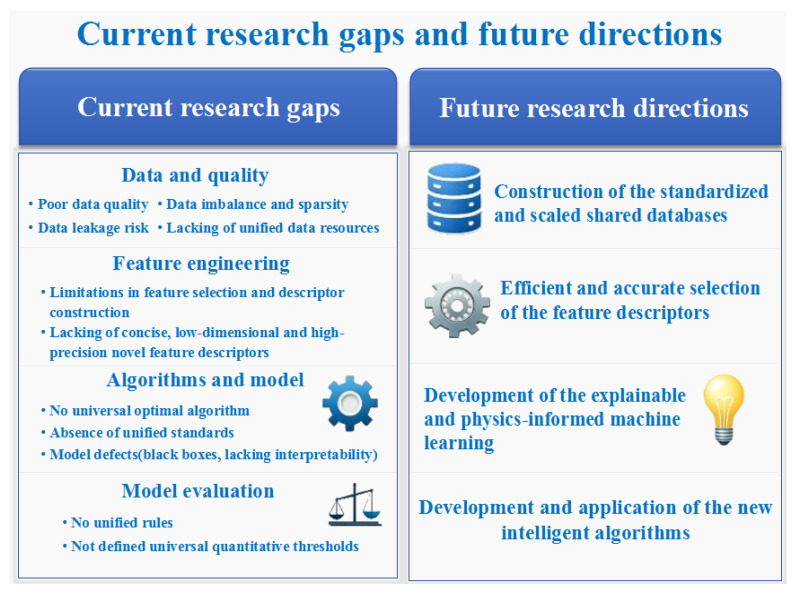
The current research gaps and future directions in the solubility of gases in ionic liquids by ML.

However, ML-based prediction still faces multiple limitations and practical challenges, with key research gaps and future directions illustrated in Figure 8. In terms of data resources, a unified, standardized and specialized database for gas capture in ionic liquids is yet to be established. Public datasets are inadequate, and data collection and calibration criteria vary significantly across studies. Most original datasets and modeling codes are undisclosed, with incomplete key experimental details, resulting in poor reproducibility of experimental and model results, and impeding the iterative upgrading and standardized development of the field.

From the perspective of model construction and technical implementation, current prediction models suffer from complex structures and the absence of universal evaluation and application standards. Divergences in algorithm selection, dataset scale and feature configuration hinder the formation of unified industrial and academic research specifications. Additionally, the inherent black box nature of machine learning models, coupled with inconsistent hyperparameter settings and data preprocessing strategies, easily causes prediction bias, overfitting and dimensionality disasters induced by low-quality data. Accordingly, machine learning still has substantial optimization potential for gas solubility prediction in ionic liquids.

(1) Construction of standardized and large-scale shared databases. This field currently lacks high-quality, publicly available experimental datasets and unified benchmark data resources. It is imperative to build a dedicated unified shared database for research of gas solubility in ionic liquids, with standardized specifications for data collection, annotation, preprocessing and storage, as well as unified criteria for experimental parameters, molecular descriptors and absorption performance indicators, to eliminate systematic errors from heterogeneous data sources. Targeted supplementation of scarce data is also required, covering novel functionalized ionic liquids and the absorption of understudied gases such as CO_2_, NH_3_ and SO_2,_ to balance the long-tail data distribution of diverse ionic liquids–gases systems. A dynamic data update mechanism should be established to incorporate the latest published data, realizing open data sharing and reusability, and underpinning the development of high-precision, generalized prediction models. Furthermore, integrating intelligent data denoising and anomaly identification algorithms enables automatic dataset cleaning and quality optimization to improve overall data reliability. The establishment of reliable, universal and standardized benchmark datasets is critical, as it can provide unified evaluation criteria for systematic performance validation of various machine learning models, serving as a key prerequisite for optimizing predictive modeling systems in this field.

(2) Efficient and accurate selection of the feature descriptors. Feature descriptors are the core determinants for enhancing model predictive performance, reducing computational overhead, and improving model interpretability, while their rational design also remains a key technical challenge in this field. Although multiple research approaches, including group contribution (GC), quantitative structure–property relationship (QSPR), molecular dynamics (MD) simulation, and machine learning, have been widely adopted to investigate gas capture in ionic liquid behavior, each individual method possesses inherent limitations. Furthermore, the research paradigm of multi-method fusion is still in its early stages of development and requires further exploration. There is an urgent demand for the development of novel descriptors that feature conciseness, low dimensionality, and high prediction accuracy. Future research can explore innovative multi-technology fusion frameworks, such as the integration of MD simulation and machine learning algorithms, or the synergistic combination of GC, MD, and machine learning methods. In addition, the unique strengths of deep learning techniques—including refined fitting capability, low prediction error, and high nonlinear fitting accuracy—can be fully exploited to further optimize the quantitative prediction of ionic liquid physicochemical properties and gas solubility performance, thereby advancing the precision and applicability of existing prediction systems.

(3) Development of the explainable and physics-informed machine learning approaches. To overcome the drawbacks of conventional black box machine learning models, interpretable, physics-embedded frameworks are urgently needed for predicting gas solubility in ionic liquids. The physicochemical adsorption rules between gas molecules and ionic liquids underpin the interpretation of solubility and gas capture capacity, and deep mechanistic cognition is also a necessary premise for QSPR-based solubility prediction. Thorough analysis of ionic liquid functional groups, skeletons, anion–cation configurations, electronic and steric effects, plus inherent adsorption behaviors, chemical reaction pathways and intermolecular forces, supports rational molecular design and precise performance forecasting of targeted gas absorbents [65,189]. Combining machine learning with molecular simulation and quantum chemistry links data-driven modeling to essential adsorption mechanisms. Matching feature construction with adsorption physicochemical nature allows screening key adsorption-related descriptors and removing redundant variables, so as to build high-accuracy feature sets guided by adsorption mechanisms. In addition, interpretability techniques (SHAP, LIME, global sensitivity analysis, causal inference) support dynamic mechanistic explanation for model outputs, quantitatively measuring how anion–cation structures, functional groups and operating parameters affect gas solubility. These interpretable models can directly guide directional structural modification of ionic liquids, accelerating the customized development of high-efficiency ionic liquid absorbents for gas separation and capture.

(4) Development and application of the new intelligent algorithms. Emerging advanced learning paradigms, including meta-learning, transfer learning, and graph neural networks, can effectively address the inherent limitations of traditional machine learning methods, such as excessive data dependence, labor-intensive feature engineering, and insufficient generalization capability. These advanced algorithms enable high-precision solubility prediction, automated feature extraction, and prominent generalization optimization under small-sample scenarios, which are highly compatible with the data characteristics of gases solubility in ionic liquid and quantum chemistry research.

**Figure 9:**
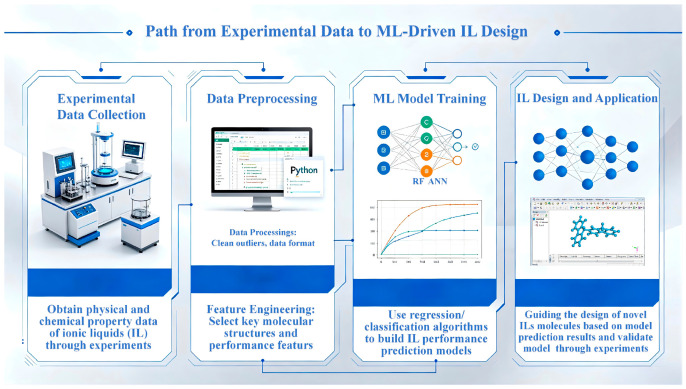
The pathway from experimental data to ML-driven IL design.

To address sparse samples and high-dimensional complex data common in this field, high-throughput computing is critical for mining hidden physicochemical rules from large datasets. Multiple schemes can improve model performance: anomaly detection to identify data noise, data augmentation to expand sample diversity and mitigate long-tail bias, and ensemble learning to strengthen model robustness and prediction stability. As an effective few-shot method, meta-learning leverages prior knowledge to adapt rapidly to new tasks, showing prominent advantages for small-sample ionic liquid–gas solubility prediction. Future work should refine advanced algorithm theories, build standardized model reliability evaluation systems, develop efficient real-time learning strategies, and unify application specifications for various algorithms. Cross collaboration among computational chemists, machine learning researchers and experimentalists is essential to build active learning and autonomous experiment platforms guided by intelligent decision-making. Such interdisciplinary research will advance automation and intellectualization in gas capture in ionic liquid studies. Overall, this framework enables a closed-loop workflow covering experimental measurement, intelligent modeling, and ML-directed customized design of ionic liquid structures, as illustrated systematically in Figure 9.

## Figures and Tables

**Table 1:** Summary of research progress in machine learning for predicting CO_2_ solubility in ionic liquids in the years 2024–2026.

Inputs	Dataset	Algorithm	Reported Performance of Best Model	Ref.
Molecular weight, Boiling point, COSMO volume, Viscosity parameter	293–363 K Total: 768 Train: 600 Test: 168	SVM	AARD% = 3.11% R^2^ = 0.8820	[122]
25 inputs	298–370 K 0–500 bars 34 ionic liquids 4700 data points	ANN 25-100-100-1	R^2^ = 0.986; MSE = 0.0008924; MAE = 0.020474	[123]
molecular fingerprints, Morgan fingerprints, ECFPs	243.2–453.15 K 0.00798–499.9 bars Total: 10,116 Train: 70%/80%/90% Test: 30%/20%/10%	GPR, LightGBM, CatBoost	CatBoost: R^2^ = 0.9925 MAE = 0.0122	[124]
T, P, and groups on cations and anions	273.15–413.15 K 9.7–6532.8 kPa Total: 3036 Train: 80% Test: 20%	GBDT, XGBoost	XGBoost: R^2^ = 0.9996 MAE = 0.00146	[125]
Pc, Tc, ω, T, P, Density, MW	Total: 1517 Train: 70% Test: 15% Validation: 15%	MLP, ANFIS, ANN	ANN: R^2^ = 0.992 RMSE = 0.04	[126]
PaDEL descriptors, T, P	243.2–453.15 K 0.00798–499.9 bars Total: 10,116 Train: 80% Test: 20%	ANN, DA-SVM	ANN: R^2^ = 0.9828 MAE = 0.0195	[127]
T, P, Functional groups of ionic liquids	243.2–453.15 K 0.00798–499 bars Total: 10,116 Train: 80% Test: 20%	ANN, LSTM, RF, GBR	ANN: R^2^ = 0.986 RMSE = 0.0273	[128]
T, P, Cation type, and Anion type	243.2–453.15 K 0.00798–499 bars Total: 10,116 Train: 80% Test: 20%	CNN, LSTM, COA, GBO	CNN-COA: R^2^ = 0.9953 RMSE = 0.0164	[129]
T, P, Chemical structure	Total: 10,341 Train: 80% Test: 20%	XGBoost, GBoost, LightGBM, CatBoost	XGBoost: RMSE = 0.014 R^2^ = 0.9967	[70]
Pc, Tc, ω, T, P	292.65–450.49 K 0.0098–72.24 MPa Total: 1517 Train: 80% Test: 20%	PSO-SVR, GWO-SVR, SSA-SVR	PSO-SVR: R^2^ = 0.9824 RMSE = 0.01881	[130]
GC, T, P	Total: 2500 Train: 80% Test: 20%	MLP, SVR, RF, GBR	GC-GBR: R^2^ = 0.9637 MSE = 0.0532	[131]
Tb, Pc, Zc, Tc, ω, P, T	271.11–453.15 K 0.00001–100.12 MPa Total: 10,368 Train: 80% Test: 20%	CatBoost, DeepGBM, DNN, KNN, LightGBM, SVR	DeepGBM: R^2^ = 0.9912 RMSE = 0.02249	[132]
SMILES, P, T, Center factor, molecular weight	300–375 K 0–5000 kPa Total: 15,666 Train: 80% Test: 20%	VAE, ANN, PSO	VAE-ANN: R^2^ = 0.9792 MAE = 0.022	[133]
Functional structure descriptor (FSD)	Total: 10,116 Train: 80% Test: 20%	LightGBM, CatBoost, GBDT, XGBoost, AdaBoost and RF	CatBoost-FSD R^2^ = 0.9945 MAE = 0.0108	[71]
Cation and anion structures	271.11–453.15 K 0.00001–105.01 MPa Total: 16,480 Train: 70% Test: 30%	MLP, RBF, RF, LSBoost	LSBoost R^2^ = 0.9962 MSE = 0.0070	[134]
P, T, atom of cation and anion and numbers	278.12–450.49 K 0.0001–1001.2 bars Total: 4397 Train: 80% Test: 20%	Extra trees, AdaBoost-SVR, CatBoost, DT, XGBoost, LightGBM	XGBoost: R^2^ = 0.999 RMSE = 0.0077	[135]
Morgan fingerprints	Total: 39,943 Train: 80% Test: 20%	GCN, GAT, GIN, GCN_GAT	GCN_GAT R^2^ = 0.971 MAE = 0.023	[136]
T, P, σ profile descriptors	243.15–453.15 K 0.798–49,990 kPa Total: 9864 Train: 80% Test: 20%	ANN, RF	ANN: R^2^ = 0.9754 MAE = 0.0257	[137]
Molecular graphs and T, P	243.2–453.15 K 0.01–106,401.3825 kPa Total: 386 25 ionic liquids	GNN(GAT, PNA, GIN, MPNN), Ensemble_ORSA, Attentive_FP, Ensemble_Stack_Ensemble_WA, Ensemble_Stack_ET	Ensemble_ORSA: R^2^ = 0.9258 RMS = 0.0621	[138]
CMDEA, CIL, T, and P_CO2_,GC, physical and chemical properties, topological indices, molecular structure	298.15–353.15 K 10–4360 kPa Total: 867 Train: 80% Test: 20%	MLP, Boost, Limelight	MLP: R^2^ = 0.9972 MSE = 0.000133	[139]
T, P, MD, GC	283.1–393.15 K 0.0004–20.006 bar Total: 2482 Train: 80% Test: 20%	Boost, Cat Boost, Boost, and Limelight	MLP: R^2^ = 0.9972 MSE = 0.000133 MD&AC-based Cat Boost R^2^ = 0.9616	[140]
Quantum chemical descriptors from COMO-RS	243.2–453.15 K 1–49,990 kPa Total: 6173 Train: 80% Test: 20%	C-DEN, MLP, P-DEN	P-DEN: R^2^ = 0.9904 RMSE = 0.0216	[141]

**Table 2:** Summary of the research progress on the application of machine learning to predict H_2_S solubility in ionic liquids.

Inputs	Dataset	Algorithm	Reported Performance of Best Model	Ref.
T, P, Tc, Pc, ω	303.15–363.15 K 0.0685–2.0168 Mpa Total: 11	RBF-ANN, BP-ANN, PSO-ANN	PSO-ANN: RMSE = 0.0141; R^2^ = 0.992	[142]
Pc, Tc, T, acentric factor	303.15–363.15 K 0.0608–2.0168 MPa Total: 465 Train: 369 Test: 96	SGB	SGB: R^2^ = 0.9995, MRAE = 0.0222	[143]
T, P, mole fraction	293.15–403.15 K 0.001–96.30 bars Total: 1282 Train: 1026 Test: 256	ELM	ELM: R^2^ = 0.999, RMSE = 0.0112	[144]
Pc, Tc, P, T, centrifugal coefficient	596.23–1366.72 K 10.05–40.46 bars Total: 1243 Train: 994 Test: 249	MLP	MLP: AARD% = 2.3292, R^2^ = 0.9982	[145]
P, Tc, Pc, molecular weight	303.15–363.15 K 0.0608–2.0168 MPa Total: 733 Train: 513 Test: 165 Validation: 55	ANN	R^2^ = 0.997, MSE = 2.463 × 10^−4^	[146]
T, P, Tc, Pc, ω	303.15–363.15 K 0.0608–2.0168 MPa	GA-LSSVM	R^2^ = 0.9976, MSE = 0.0082	[147]
T, P, Tc, Pc, ω	303.15–363.15 K 0.039–2.017 MPa	ANN	R^2^ = 0.9987	[78]
P, Pc, T, Tc	298–403 K 0.069–9.630 MPa Total: 496 Train: 392 Test: 104	ANN	All predicted points showed deviations lower than 12.6%	[148]
Tc, P, Pc, T, molecular weight of pure ionic liquids	298.15–363.15 K 0.0582–9.63 MPa Total: 664 Train: 554 Test: 110	MLP, ANFIS, RBF	MLP-ANN: R^2^ = 0.9951, MSE = 0.000117	[149]
acentric factor, Tc, Pc	293.15–403.15 K 0.001–96.30 × 10^−5^ Pa Total: 1282 Train: 962 Test: 320	LSSVM	LSSVM: R^2^ = 0.996, AARD = 2.63%, RMSE = 0.011	[150]
chemical structure, T, P	293.15–403.15 K 0.001–96.3 bars Total: 1516 Train: 1212 Test: 304	CNN, RNN, DBN	Models: R^2^ > 0.99, RMSE < 0.01	[151]
T, P, Tc, Pc, acentric factor, boiling temperature, molecular weight	293.15–403.15 K 0.001–96.3 bars Total: 1516 Train: 80% Test: 20%	GP, DBN, XGBoost	XGBoost: RMSE = 0.002, R^2^ = 0.99	[152]
MFI, MD, MD-MFI	-	DNN, RF	DNN: R^2^ = 0.9923, RMSE = 0.0151	[143]
T, P, acentric factor, Pc, Tc	298.15–403.15 K 0.58–96.3 bars Total: 792 Train: 673 Test: 119	ANFIS, LSSVM, RBF, MLP, Cascade, GRNN	LSSVM: R^2^ = 0.9980, RMSE = 0.0108, MSE = 0.0001	[153]
Electrostatic potential surface (SEP)	293.15–403.15 K 0.001–96.3 bars Total: 1318 Train: 1055 Test: 263	ELM	R^2^ = 0.994, AARD% = 5.68%	[154]
T, P, mass concentration, mixture’s apparent molecular weight	298.434.5 K 13–9319 kPa Total: 670 Train: 88% Test: 9% Validate: 3%	LSSVM	AARD% = 2.6%, R^2^ > 0.9954	[155]
Sσ-profile	293.15–403.15 K 0.001–97.3 bars Total: 1282 Train: 80% Test: 20%	ELM	AARD% = 3.73%, R^2^ = 0.998	[156]
SMILES, graph descriptors, hybrid EState-VSA descriptors	298.15–403.15 K 0.11–96.3 bars Total: 722 Train: 80% Test: 20%	GPR, XGBoost, RF, SVM	GPR: R^2^ = 0.9918, RMSE = 0.0147	[157]
Multicomponent Graph Representation	Total: 1516	GPR, PEMC D-MPNN, XGBoost, RF, SVM, DBN, DJINN, RNN, GMDHGP	PEMC D-MPNN: R^2^ = 0.9964, RMSE = 0.0099	[158]

**Table 3:** Research progress on machine learning methods for the prediction of NH_3_ solubility in ionic liquids.

Inputs	Dataset	Algorithm	Reported Performance of Best Model	Ref.
Tc, Pc, centrifugal factor, T, P	282.2–372.8 K 0.01–5.01 MPa Total: 320 Train: 256 Test: 64	SVM, MLP, RF, DNN	MLP: MAE = 0.0004, R^2^ = 0.992	[164]
Electrostatic potential surface area, T, P	282.2–372.8 K 0.3996–50.07 bars Total: 502 Train: 402 Test: 100	ELM	ELM: R^2^ = 0.9937 AARD% = 2.95%	[166]
T, P, HBD molar dose	298.2–353.2 K 2.3–573.2 kPa Total: 793 Train: 674 Test: 119	MLP, WNN, GRN, RBF, CFFNN, GRNN	CFFNN: R^2^ = 0.995 MAE = 7.44	[167]
Molecular weight of ionic liquids, Tc, Pc, T, P	282.2–372.8 K 0.1–5.007 MPa Total: 352 Train: 264 Test: 88	PSO-ANFIS, MLP	MLP: R^2^ = 0.9967, AARD% = 1.23697	[168]
Chemical structure (molar concentration, HBA and HBD type, water dosage), T, P	298.2–353.2 K 0.1–573.2 kPa Total: 1356 Train: 1085 Test: 271	ANFIS	ANFIS: MSE = 0.216, R^2^ = 0.998	[169]

**Table 4:** Summary of research progress of gases (SO_2_, N_2_O, O_2_, H_2_, CH_4_) solubility in ionic liquids using machine learning methods.

Inputs	Molecule	Dataset	Algorithm	Reported Performance of Best Model	Ref.
T, P, ionic liquids’ critical properties	N_2_O	298 K 1 bars Total: 25	ANN, SVM, LSSVM	SVM: R^2^ = 0.9970 RMSE = 0.0104	[175]
T, P, ionic liquids’ critical properties	N_2_O	283.15–373.55 K 0–301.00 bars Total: 533 Train: 426 Test: 107	CFNN, GEP, RBF	CFNN: R^2^ = 0.9994 RMSE = 0.0047	[176]
T, Tc, P, ω, Mw, Pc	N_2_O	303.85–1314.1 K 0.000837–301 bars Total: 25	DBN, Cat-boost, ELM, XGB	XGB: R^2^ = 0.9999 RMSE = 0.0016	[177]
T, Mw, Pc, Tc, P	N_2_O	600.542–1586.735 K 0.000288–301 bars Total: 25	SVM, ANN, LSSVM	LSSVM: R^2^ = 0.9970 RMSE = 0.1640	[177]
acentric factor, Pc, P, T, Tc	N_2_O	643.18–1586.7 K 850–4509 kPa Total: 521 Train: 80% Test: 20%	LR Voting, FFNN	FFNN: R^2^ = 0.9981 MSE = 0.00002	[175]
T, P, and 2D molecular descriptors of ionic liquids	SO_2_	283.15–348.15 K 0.005–3.017 MPa Total: 340 Train: 80% Test: 20%	CNN	R^2^ = 0.983	[172]
P, T, critical compressibility, centrifugal coefficient	SO_2_	282.80–348.20 K 0.005–3.017 MPa Total: 232 Train: 140 Test: 92	LSSVM	Total: R^2^ = 0.991 Train: R^2^ = 0.995 Test: R^2^ = 0.986	[171]
ionic liquids molecular weight, water content, P, T	SO_2_	283.1–373.6 K 1–30,100 kPa Total: 374 Train: 299 Test: 75	RNN, ELM, MLP, RBF, CFF	MLP: R^2^ = 0.97936 MSE = 1.13 × 10^−3^ MAPE = 4.76%	[179]
T, Chemical structure of ionic liquids, P	SO_2_	283.015–413.331 K 13.92745–49.63574 bars Total: 480 Train: 384 Test: 96	LSSVM	R^2^ = 0.9978 MSE = 0.0103	[174]
T, Pc, Tc, P,ω	SO_2_	282.80–348.20 K 0.005–3.017 MPa	ANN	R^2^ = 0.9861 MSE = 0.0217	[170]
(I) chrmical structure (II): thermodynamic feature	H_2_	293.15–353.20 K 0.1–573.2 kPa	RF, SVR, MARS, AdaBoost, DBN	DBN: (I) RMSE = 0.00106 R^2^ = 0.9991 (II) RMSE = 0.00066 R^2^ = 0.9996	[180,181]
(I): chemical structure (II): thermodynamic feature	O_2_	278.2–453.1 K 0.433–552 bars Total: 580 Train: 464 Test: 116	XGB, DBN, MARS, CatBoost	(I) DBN: R^2^ = 0.9976 RMSE = 0.00341 (II) XGB: (II) RMSE = 0.00095 R^2^ = 0.9998	[182]
σ-profiles, T, P	N_2_	283.15–413.2 K 0.0002–97.2 bar Total: 743	GBR, RF	RF: 0.9983 GBR: 0.9999 (train set)	[183]
acentric factor, P, Pc, T, Tc	CH_4_	283.2–363.5 K 0.0469–684.84 bars Total: 385 Train: 80% Test: 20%	MLP	MAD = 3.59% (train set) MAD = 3.57% (test set)	[184]
T, ω, P, Pc, Tc, Mw	CH_4_	293.15–449.12 K 0.400–16.105 MPa Total: 440 Train: 396 Test: 22	CSA-LSSVM, PSO-ANFIS, Hybrid-ANFIS, MLP-ANN, PSO-ANFIS, CSA-LSSVM	CSA-LSSVM AARD = 2.29%	[185]
T, ω, P, Pc, Tc, Mw	CH_4_	297.15 K 22 bars	PSO-ANFIS, CSA-LSSVM	CSA-LSSVM AARD = 3.04%	[105]
T, ω, P, Pc, Tc, Mw	CH_4_	293.15–453.15 K 0.2020–120.49 bar	ANN	AARD = 17.33	[186]
	CH_4_	283.05–453.15 K 0.0158–201.64 bars	ANN, SVM, LSSVM	ANN AARD = 16.17	
	C_2_H_6_	283.05–453.15 K 0.0158–201.64 bars Train: 75% Test: 25%	ANN, SVM, LSSVM	LSSVM AARD = 34.44	
	C_3_H_8_	283.15–368.4 K 0.0002–130.7 bars Train: 75% Test: 25%	ANN, SVM, LSSVM	SVM AARD = 16	
T, ω, P, Pc, Tc, Mw	C_4_H_10_	279.98–339.97 K 0.999–12.182 bars Train: 75% Test: 25%	ANN, SVM, LSSVM	ANN AARD = 2.8	[187]
T, Mw_ionic liquids, P, Pc_ionic liquids, Tc_ionic liquids, ω_ionic liquids, Mw_gas_, Tc_gas, Pc_gas, gas kinetic diameter, ω_gas_	CO_2_, CH_4_, H_2_S, N_2_O, SO_2_, CO	Train: 75% Test: 25%	MLP-ANN, Hybrid-ANFIS, PSO-ANFIS, CSA-LSSVM	MLP-ANN: R^2^ = 0.9945 MSE = 0.0173	[105]
T, Mole fraction of IL, P, Mole ratio of CO_2_ to second gas in feed, IL Mw, Pc, Tc, ω and second gas Tc, Mw, Pc	CO_2_, H_2_S, SO_2_, CH_4_, H_2_, N_2_O	282.4–543.15 K 0.050–971 bars Total: 40	LSSVM	R^2^ = 0.9999 MSE = 0.0005	[89]
T, P, Mw_gas_, ω_gas_	C_2_H_6_, CH_4_, O_2_, N_2_, Ar, CO	294.8–573 K 0.105–12.866 MPa Total: 11	ANN		[188]
X_CH4_, X_CO2_, %_CH4_ (feed), %_CO2_ (feed), T, Tc, Pc, ω	CO_2_, CH_4_	283.0–344.3 K 0.2189–0.9853 bars	CSA-LSSVM, PSO-ANFIS	CSA-LSSVM: R^2^ = 0.9846	[174]

## Data Availability

No new data were created or analyzed in this study. Data sharing is not applicable to this article.

## Supplementary Materials

The following supporting information can be downloaded at https://www.mdpi.com/article/10.3390/molecules31132293/s1, Table S1: Table of advantages and disadvantages of machine learning algorithms and Table S2: Summary of research progress on the CO_2_ solubility of ionic liquids using machine learning before 2024.

## Symbols and Nomenclature

RF	Random forests
DT	Decision trees
SVM	Support vector machines
EL	Ensemble learning
KNN	K-Nearest neighbors
NN	Neural networks
XGBoost	Extreme gradient boosting
NB	Naïve Bayes
NNB	Neural naïve Bayes
ANN	Artificial neural network
ANFIS	Adaptive neural fuzzy inference system
MLP	Multilayer perceptron
SRK	Soave Redlich Kwong
PR	Peng Robinson
GPR	Gaussian Process Regression
CatBoost	Categorical Boosting
SVR	Support vector regression
LSSVM	Least Squares Support Vector Machine
GA	Genetic algorithm
CART	Classification And Regression Tree
DNN	Deep Neural Network
CNN	Convolutional Neural Networks
MLP ANFIS	Multilayer perceptron—adaptive neural fuzzy inference system
CHPSO-ANFIS	Conjugate Hybrid and Particle Swarm Optimization—adaptive neural fuzzy inference system
CSA-LSSVM	Simulated annealing algorithm-least squares support Vector machine
RBF-NN	Radial Basis Function Neural Networks
MLP	MultiLayer Perceptron
CMIS	Committee machine intelligent system
MLP-NN	Multilayer perceptron neural network
PSO-RBF	Radial basis function neural network optimized by particle swarm optimization method
Hybrid-ANFIS	Adaptive neuro-fuzzy inference system trained by hybrid method
CSA-LSSVM	Least square support vector machine optimized by coupled simulated annealing
MLP-NN	Multilayer perceptron Artificial Neural Network
RBF-ANN	Radial Basis Function Artificial Neural Network
ANFIS	Adaptive Neuro-Fuzzy Inference System
RBF-ANN and ANFIS	Radial Basis Function Artificial Neural Network and Adaptive Neuro-Fuzzy Inference System
GA-MLR and LSSVM	Genetic algorithm-multi linear regression and least-squares support vector machines
MLR	Multi linear regression
PLSR	Partial Least Squares Regression
CTREE	Conditional Inference trees
RBF	Radial basis function
BPNN	Back-propagation neural network
GRNN	General regression neural network
CAS-LSSVM	Least square support vector machine optimized by coupled simulated annealing
PSO-ANFIS	Particle Swarm Optimization-Adaptive Neuro Fuzzy Inference System
MLP-ANN	Multi-Layer Perceptron Artificial Neural Network
ELM	Extreme learning machine
RNN	Recurrent Neural Network
GMDH	Group method of data handling
GEP	Gene expression programming
MLP-BR	Multilayer perceptron- Bayesian Regularization
MLP-LMA	Multilayer perceptron Levenberg–Marquardt
MLFNN	Multilayer feed-forward neural network
MLP	Multi-Layer Perceptron
CFNN	Cascade feed-forward neural networks
GRNN	Generalized regression neural network
MP-MCTS	Multi-player Monte Carlo tree search
FLR	Linear Regression
GIN	Graph isomorphism network
GAT	Graph attention network
GCN	Graph convolutional network
FFNN	Feed-forward neural network
RBFNN	Radial-based function neural network
SVM	Support vector machine
SVR	Support vector regression
PSO	Particle swarm optimization
GWO	Grey Wolf Optimization
SSA	Sparrow Search Algorithm
XGB	Extreme Gradient Boosting
CatBoost	Categorical Boosting
SDVAE	Syntax-directed variational autoencoder
DeepFM	Deep factorization machine
GBPSO	Gradient-based particle swarm optimization
GBR	Gradient-Boosted Regression
LightGBM	Light Gradient Boosting Machine
GBDT	Gradient Boosting Decision Tree
DA-SVM	Support vector machine optimized with dragonfly algorithm
LSTM	Long Short-Term Memory
COA	Cuckoo optimization algorithm
GBO	Gradient-based optimization
GBoost	Gradient Boosting
PSO-SVR	Particle swarm optimization-support vector regression
GWO-SVR	Grey Wolf Optimization-support vector regression
SSA-SVR	Sparrow Search Algorithm-support vector regression
DeepGBM	Deep Gradient Boosting Machine
VAE	Variational autoencoder
AdaBoost	Adaptive Boosting
LSBoost	Least Squares Boosting
Extra trees	Extremely Randomized Trees
AdaBoost-SVR	Adaptive Boosting-Support vector regression
GCN-GAT	Graph convolutional network—graph attention network
GNN	Graph Neural Network
PNA	Principal Neighbor Aggregation
MPNN	Message-passing neural network
Ensemble_ORSA	Ensemble-Outlier-removed statistical averaging
Attentive-FP	Attentive-Fingerprint
Ensemble_Stack_Ensemble_WA	Ensemble_Stack_Ensemble_Weighted averaging
Ensemble_Stack_ET	Ensemble_Stack_extremely randomized tree
C-DEN	Component based-Deep Neural Network
P-DEN	Point-based-Deep Neural Network
SGB	stochastic gradient boosting
DBN	Deep belief networks
GP	Genetic programming
GA-LSSVM	Genetic algorithm—least square support vector machine
BP-ANN	Back propagation-artificial neural network
PSO-ANN	Particle swarm optimization-artificial neural network
PEMC	Pretraining enhanced multicomponent
D-MPNN	Directed message passing neural network
DJINN	Deep neural network initialization with decision trees
GMDH-GP	Group method of data handling -genetic programming
LASSO	Least absolute shrinkage and selection operator
CFF	Cascade feedforward
MARS	Multivariate adaptive regression splines
CFFNN	Cascaded Feed-Forward Neural Network
WNN	Wavelet Neural Network
GMM	Gaussian mixture model
PCA	Principal component analysis
Pc	Critical pressure
RFE	Recursive Feature Elimination
Mw	Molecular weight
FSD	Functional structure descriptors
P	Operating pressure
T	Operating temperature
Zc	Critical compressibility factor
ω	Eccentricity factor
Tc	Critical temperature
GC	Group Contribution
FP	Molecular fingerprint
SMILES	Simplified Molecular Input Line Entry System
RFE	Recursive Feature Elimination
MRAE	Average relative absolute error
AARD	Average absolute relative deviation
STD	Standard deviation
AAD	Average absolute deviation
MAPE	Mean absolute percentage error
ARD	Average relative deviation
RMSE	Root means square error
MAE	Mean absolute error
MSE	Mean squared error
R^2^	Correlation coefficient
