# Supplementary material for: Machine Learning for Gas Capture in Ionic Liquids: Current Status and Future Trends
Source: Molecules. 2026 Jul 1;31(13):2293. doi: 10.3390/molecules31132293 (PMC13363378; doi:10.3390/molecules31132293)
Supplement: Supplementary file 1 [file molecules-31-02293-s001.zip › molecules-4381722-supplementary.pdf]

Supporting Information

Machine Learning for Gas Capture in Ionic Liquids: Current Status and Future Trends

Guocai Tian *, Zhiqing Hu, Ranran Geng

State Key Laboratory of Complex Non-Ferrous Metal Resource Clean Utilization, Faculty of Metallurgical and Energy Engineering, Kunming University of Science and Technology, Kunming 650093, China

*Correspondence: tiangc@kust.edu.cn; Tel.: +86-13759558375

Content of Supporting Information

Table S1 The advantages and disadvantages of machine learning algorithms for gas solubility in ILs.

Table S2 Summary of research progress on the CO_2 solubility of ionic liquids using Machine Learning before 2024.

Table S1 The advantages and disadvantages of machine learning algorithms for gas solubility in ILs.

Algorithm	Full name	Advantages	Disadvantages
ANFIS	Adaptive-Network-Based Fuzzy Inference System	Nonlinear function modeling. Online identification and control of nonlinear components. Predicting chaotic time series.	Can only output univariate results. Difficulty in parameter selection.
ANN	Artificial Neural Network	High classification accuracy. Strong parallel capability. High robustness and fault tolerance. associative memory.	Complex parameter tuning. The credibility and acceptability of the results are low. Long study time.
BPNN	Backpropagation Neural Network	Simple and easy to implement. Low computational complexity and strong parallelism.	Slow convergence speed. Low learning efficiency.
BRANN	Bayesian regularized artificial neural network	Prevent overfitting. High robustness. Simplify model selection.	High computational complexity. The difficulty of parameter adjustment is high. Sensitive to initial conditions.
CART	Classification and Regression Trees	Intuitive and easy to understand. Can handle both classification and regression problems simultaneously. Capable of processing high-dimensional data. High computational efficiency.	Easy to overfit. Sensitive to noise and outliers. Poor stability.
CatBoost	Categorical Boosting	Excellent performance. Better robustness and versatility. Easy to use and more practical.	High hardware requirements. Difficulty in adjusting hyperparameters.
CFF	Cyclic first adaptation algorithm	Quantitatively measure the characteristics of the curve. Improve optimization accuracy.	Affected by specific application scenarios, data distribution, and hardware conditions.
CNN	Convolutional Neural Networks	Local perception. Parameter sharing. Hierarchical feature extraction. High computational efficiency.	Fully connected layer redundancy. Insufficient understanding of features. High demand for training data.
CTREE	Classification and Regression Trees	Easy to understand and explain. Low requirements for data preprocessing. Capable of handling non-linear relationships. Has certain robustness to outliers and missing values. Suitable for feature selection.	Easy to overfit. Sensitive to noise. Poor stability.

DBN	Deep Belief Network	High flexibility. Less convergence time. Parallel computing. Easy to expand.	Data is limited by dimensions. Complex parameter selection. Low learning efficiency.
DCNN	Deep Convolutional Neural Networks	Powerful feature extraction capability. High flexibility and scalability. Strong robustness.	Requires a large amount of training data. High model complexity. Easy to overfit. Lack of interpretability.
DNN	Deep Neural Networks	The model is simple. Fast learning speed. Less time consumption.	The activation function is simple. Unable to learn complex nonlinear models.
DT	Decision Tree	Easy to understand and explain. Simultaneously processing nominal and numerical data. Handling samples with missing attributes and irrelevant features. Fast calculation speed.	Easy to overfit. Easy to overlook the interdependence of attributes in the dataset. Has a propensity for attribute selection.
ELM	Extreme Learning Machine	Fast learning speed. Strong generalization ability.	The results are unstable. Poor non-linear ability.
FFANN	Feed-Forward Artificial Neural Network	Universal approximation ability. Parallel processing capability. Strong learning ability.	High demand for computing resources. Parameter tuning is complex. Easy to fall into local optima. Lack of interpretability.
FNN	Feedforward Neural Network	High computational efficiency. Easy to implement and train. Strong universality.	Can only handle static data. Easy to fall into local optima. Sensitive to parameters.
GA	Genetic algorithm	Strong global search capability. High parallelism. Strong flexibility and applicability.	Complex programming implementation. High sensitivity of parameters. Slow convergence speed.
GAT	Graph Attention Networks	Having an attention mechanism. Handling complex graph structures. Efficient computing.	Oversmoothing problem. Restricted receptive field. Generate redundant calculations.
GBM	Gradient Boosting Machine	High predictive accuracy. Easy to handle non-linear relationships. High robustness. Can evaluate the importance of features. Strong scalability.	Long training time. Complex hyperparameter adjustment. Easy to overfit. Not suitable for high-dimensional sparse data.
GBT	Gradient Boosting Tree	Superior predictive performance. High flexibility and robustness. Easy to understand and explain.	High computational complexity. Easy to overfit. Sensitive to parameters.
GCN	Graph Convolutional Networks	Powerful feature extraction capability. High flexibility and scalability.	High computational complexity. Overfitting risk.
GNN	Graph Neural Networks	Process graph structure data. Capture global and local information. Strong generalization ability.	High computational complexity. Complex parameter adjustment. Low interpretability.
GPR	Gaussian Process Regression	High flexibility. Predictive uncertainty. Automatic learning.	High computational complexity. Affected by dimensions. Restricted by data distribution.

GRNN	General Regression Neural Network	Used for non parametric estimation methods. Fast calculation speed. Strong handling of noise and nonlinear problems.	High requirements for data sample size. High complexity in processing high-dimensional data. Limited global approximation ability.
K-means	K-means Clustering	Simple and easy to implement. Fast calculation speed. Processing big datasets.	High requirements for data types. Slow convergence. Sensitive to noise and outlier data.
K-NN	K-Nearest Neighbors	Separable and regrettable. Can be used for nonlinear classification. Online data processing. The theory is simple and easy to implement.	Poor effectiveness in handling imbalanced samples. Requires a large amount of memory. For datasets with large sample sizes, the computational workload is relatively high. When the sample is imbalanced, the prediction deviation is relatively large.
KRR	Kernel Ridge Regression	Nonlinear modeling ability. Ability to resist noise. Easy to implement and adjust parameters.	High computational complexity. High memory consumption. Complex kernel function selection.
LASSO	Least absolute shrinkage and selection operator	Strong feature selection ability. Easy to explain and understand. Dealing with multicollinearity problems.	Excessive compression of non-zero coefficients may occur.
LightGBM	Light Gradient Boosting Machine	Faster training speed and efficiency. lower memory usage. higher accuracy. support for parallelization of large-scale data processing.	Easy overfitting. Sensitive to noise.
LR	Logistic Regression	Simple and easy to understand. High computational efficiency. Strong interpretability. Parallelizable.	Easy to underfit. Strong dependence on feature engineering. Sensitive to outliers and noise. Not suitable for handling multi classification problems.
LSSVM	Least Squares Support Vector Machine	High predictive accuracy. Fast convergence. Avoid difficulties in parameter selection. Processing small samples.	Lack of sparsity. Sensitive to noise. High computational cost.
MARS	Multivariate Adaptive Regression Splines	Processing high-dimensional datasets. Handling classification and regression issues. Automatically select appropriate basis functions and interaction effects. High computational efficiency.	Not applicable to datasets with outliers. Easy to overfit. Not suitable for discontinuous independent variables.
MLFNN	Multi-Layer Feedforward Neural Network	Strong mapping ability. Self learning and adaptive abilities. Parallel processing. Strong generalization ability and flexibility.	Can only handle static input. Easy to overfit. Easily affected by local minima. Unable to process sequence data.

MLP (MLPNN)	Multilayer Perceptron	Strong non-linear fitting ability. Good generalization ability. Suitable for large-scale data processing and parallel computing.	Easy to fall into local minima. The parameter tuning process is relatively complex. The learning speed of complex tasks is relatively slow. Consuming a large amount of computing resources and storage space.
NLR	Neural Network Regression	Strong non-linear modeling ability. Strong adaptability and flexibility. Strong robustness to noise and outliers.	High model complexity and computational cost. Complex parameter tuning. Poor interpretability. Overfitting.
PLSR	Partial Least Squares Regression	Processing high-dimensional data. Processing data with multicollinearity. Can handle multiple dependent variables simultaneously.	High quality data requirements. Large computational load, requiring high performance.
PSO	Particle Swarm Optimization	The algorithm has strong universality. Simple and easy to implement. Collaborative search. Fast convergence speed. Algorithms do not require high computer memory and CPU requirements.	Poor local search ability and insufficient search accuracy.
RBM	Restricted Boltzmann Machine	Automatic learning of input features. High flexibility. High training efficiency	Computational complexity. Model instability. Representation ability may be limited by its structure
RBF (RBFNN)	Radial Basis Function	Excellent non-linear mapping ability. Fast training speed. Stable performance.	Difficulty in selecting suitable RBF centers. poor network generalization ability, and high computational complexity.
RF	Random Forest	Processing high-dimensional data. Determine the importance of features and the mutual influence between different features. Not easily overfitting. The training speed is relatively fast. Easy to implement. Balance error.	Overfitting may occur in classification or regression problems with high noise levels. Affected by data of attributes with different values.
RFR	Random Forest Regression	Powerful predictive performance. The ability to process high-dimensional data. High robustness to missing and outliers. Easy to implement and adjust parameters.	High computational cost. Possible overfitting of noise. Relatively weak explanatory power.
RNN	Recurrent Neural Network	Processing sequence data. Having memory ability. Parameter sharing. Turing Complete.	Having short-term memory problems. Unable to handle long input sequences. High consumption.

SGB	Stochastic Gradient Boosting	Reduce the risk of overfitting. High computational efficiency. High parallelization ability. High predictive performance.	The complexity of parameter tuning is high. Sensitive to noise. Low interpretability.
SVM	Support Vector Machine	Dealing with high-dimensional problems. Solving machine learning problems with small samples. Dealing with the interaction of nonlinear features. No local minimum problem. No need to rely on the entire data. Comparison of generalization ability.	When there are many samples, the efficiency is not high.The explanatory power of high-dimensional mapping for kernel functions is not strong.Conventional SVM only supports binary classification Sensitive to missing data.
SVR	Support Vector Regression	High generalization ability. Suitable for nonlinear problems. Good robustness.	Complex parameter tuning.High computational cost.Sensitive to the selection of kernel functions. Relatively weak explanatory power.
WNN	Wavelet Neural Network	A solid theoretical foundation. Avoid local optima.Strong ability to learn and generalize functions.Widely applicable.	Poor accuracy instability.early maturity.high computational complexity.
XGBoot (XGB)	eXtreme Gradient Boosting	High precision. Strong flexibility. Support parallelism.	Parameter tuning is complex.High memory consumption.Easy overfitting.

Table S2 Summary of research progress on the CO_2 solubility of ionic liquids using Machine Learning before 2024.

Inputs	Data set	Algorithm	Best model	Ref
Pc, Tc, ω, p, T	304.25-636 k<br>0.1-598 bars<br>Total: 1128<br>24 ILs	ANN	R^2 = 0.995<br>RMSE = 0.05<br>AAD% = 3%	[67]
T, Total concentration of the mixture, Partial pressure, Apparent Molecular weight	298.15-453.15 K<br>0.604-19.914 kPa<br>Total: 632	ANN<br>4-8-4-1	R^2 = 0.995<br>MRE = 2.796<br>MSE = 1.406E04	[74]
P, Percentage weight of ethanol&IL of [EMIM][Tf_2N] ,T	313.2-333.2 K<br>0-7 MPa<br>Total: 70<br>1 IL	ANN<br>4-15-1	R^2 = 0.994<br>AAD = 4.94<br>MRE = 2.796	[75]
Apparent molecular weight, Pc, Tc, Partial pressure of CO_2, Overall mass concentration,T	298-434.15 K<br>13-9319 kPa<br>Total: 114	ANN	MRE = 4.143,<br>MSE = 1.011E-4<br>R^2 = 0.998	[76]
P, T, center factor, molecular weight	298-373.15 K<br>0.0097-100.2 MPa<br>Total: 728<br>Train: 546<br>Test: 182	MLP ANFIS	MLP:<br>R^2=0.9972<br>MSE=0.000133	[68]
Water content, IL content, P, T	25-60 ℃<br>0-50 bars<br>Total: 54<br>1 IL	ANN<br>4-14-1	R^2 = 0.998<br>SD = 0.005<br>MSE = 2.22E-05	[77]
P, T, Pc, Tc, ω	278.5-450.49K<br>0.01-97 MPa<br>Total: 2930<br>39 ILs	ANN<br>5-23-1	R^2 = 0.9907;<br>MSE = 0.00115;<br>AAD = 3.58%	[78]
T, mole fraction of H_2O, gua, MDEA	303-323 K<br>Total: 77<br>Train: 42<br>Test: 29<br>Validate: 16	ANN	R^2=0.996<br>RMSE=0.0004<br>AAD=0.0271	[79]
T, concentration of PZ, partial pressure of CO_2,	298-343 K<br>0.032-1487 kPa<br>Total: 390	CHPSO-ANFIS and CSA-LSSVM	CHPSO-ANFIS:<br>R^2 = 0.988;<br>RMSE = 0.0458;<br>AARD% = 4.29	[80]

T, P , critical temperature & pressure, molecular weight, acentric factor	298-373 K 0.0097-100 MPa Total: 728 14 ILs	RBFNN LSSVM MLP CMIS	CMIS: R^2=0.998454; AARD = 7.152468 RMSE = 0.008246	[81]
T, P, Tc, Pc, Acentric factor	271-453 K 0.0097-100 MPa Total: 5332 66 ILs	CART	R^2 = 1 ARD% = 0.01 AARD% = 0.06	[72]
Pc,Tc, P, T, Centrifugal factor	271-453 K 0.0097-100 MPa Total: 5330 Train: 4797 Test: 533	CART	R^2=1 AARD%=0.04 (test sets)	
T, P , IL molecular weight, Number of hydrogen Atoms in anion part, Number of carbon atoms in cation part & in anion part,number of IL sulphur, Nitrogen, Oxygen, Chlorine, Fluorine & boron atoms	278.47-450.5 K 0.01-97 MPa Total: 2930 39 ILs	MLP-NN, PSO-RBF, Hybrid-ANFIS, CSA-LSSVM	CSA-LSSVM: R^2= 0.9942; RMSE = 0.0172; AARD% = 1.98	[82]
Temperature, Pressure, Critical pressure & Temperature, Acentric factor	271.1-453 K 0.0097-100.2 MPa Total: 5368 67 ILs	LSSVM, MLP-ANN, RBF-ANN and ANFIS	LSSVM: R^2 = 0.9942; RMSE = 0.018815	[83]
Average bonding Information content, Number of single bonds (Nsb), the Image of the Onsager-Kirkwood solvation energy	21 ILs	GA-MLR and LSSVM	MLR: R^2 = 0.966; RMSE = 0.016	[84]
Image of the Onsager-Kirkwood solvation energy, Number of single bonds	Total: 21 ILs Train: 16 ILs Test: 5 ILs	MLR, GA,LSSVM	LSSVM: R^2=0.962 RMSE=0.015 (total sets)	[85]
Semi empirical (PM6) electronic, Thermodynamic properties	271-453K 0.001-95.7 bars Total: 10848 Train: 5424 Test: 5424	PLSR, CTREE, RF	RF: R^2=0.95 RMSE<0.05(train sets)	[86]

Chemical structure of PILs, T, P	298.15-348.15 K<br>1.11-79.85×10^5 Pa<br>Total: 350<br>Train: 70%<br>Test: 25%<br>Validate: 5%	ANN, ANFIS	ANN:<br>R^2= 0.9999<br>MSE= 0.0158	[87]
T, P, Tc, Pc, ω, Mw	293-363 K<br>0.01-49.990 MPa<br>Total: 184<br>Train: 70%<br>Test: 30%	ANN	R^2=0.9994<br>MSE=2.9336×10<br>-5	[88]
T, P, Chemical structures	294.5-573 K<br>0.105-12.866 MPa<br>Total: 513<br>Train: 128<br>Test: 385	RBF, LSSVM	LSSVM:<br>AARD%=0.271<br>R^2=1.00	[73]
P, T, Mole fraction of ILs,	298-333 K<br>0.069-47.52 kPa<br>Total: 105<br>Train:<br>70%/80%/90%<br>Test:<br>30%/20%/10%	LSSVM	LSSVM:<br>MRE=0.7545<br>R^2=1.0000	[89]
Concentration of PL and MEA, P, T	302-363.7 K<br>0.4-12.8 MPa<br>Total: 1119<br>Train: 783<br>Test: 336	BPNN,<br>GRNN	BPNN:<br>RMSE<0.02<br>GRNN:<br>RMSE<0.12<br>(train set=90%)	[90]
Molar fractions of CH_4 and CO_2, T, Pc	278.15-450.49 K<br>0.25-100.12 MPa<br>Total: 1386<br>Train: 970<br>Test: 208	CSA-LSSVM,<br>PSO-ANFIS	CSA-LSSVM:<br>R^2= 0.9815;<br>%AARD =<br>3.4568;<br>MSE = 0.1133	[91]
Pc, Tc, ILs' molecular structure	243.15-410.90 K<br>0.010-100.120<br>MPa<br>Total: 544<br>Train: 70%<br>Test: 15%<br>Validate: 15%	MLP-ANN	MLP-ANN:<br>R^2=0.9987<br>MSE=0.6293	[92]
T, Tc,P, Pc, Mw, ω	294.5-573 K<br>0.105-12.866 MPa<br>Total: 513<br>Train: 128<br>Test: 385	BPNN, SVM,<br>ELM, LFM	LFM:<br>R^2=0.9985<br>MAE=0.0060	[93]

T, Tc,P, Pc, Mw, ω	Total: 728	DNN, CNN, RNN, XGBoost	CNN: R^2 = 0.999; RMSE = 0.206; RNN: R^2 = 0.988; RMSE = 0.651;	[94]
Model 1: T, Tc,P, Pc, Mw, ω<br>Model 2: g Chi_G/D, Homo-Lumo fraction, Disps, 3D, SpMax_RG, P, T	283-450.2 K<br>0.01-95500 kPa<br>Model 1: Total: 1655<br>36 ILs	Decision Tree, Random Forest, LSSVM, MLR	DT: R^2 = 0.94; AARD = 21.24%<br>RF: R^2 = 0.96; AARD = 12.05%	[95]
T, Tc,P, Pc, Mw, ω	283.15-373.35 K<br>99.8-85800.0 kPa<br>Total: 1155<br>24 ILs	GMDH, RBF-NN	RBF-ANN: R^2 = 0.9879; RMSE = 1.9706; AARD% = 4.43	[96]
T, Tc,P, Pc, Mw, ω	298-373.15 K<br>0.01-100.12 MPa<br>Total: 744<br>13 ILs	GEP, MLP-BR, MLP-LMA	MLP-BR: RMSE = 0.0138; R2 = 0.9951	[97]
T, Tc,P, Pc, Mw, ω	278.12-450.49 K<br>0-100.12 MPa<br>Total: 1468<br>9 ILs	Selective ensemble model	R^2= 0.9981; MAE = 0.0049 RMSE = 0.0096	[98]
ILs' molecular structure, T, P	243.2-453.15 K<br>0.00798-499.9 bars<br>Total: 10116<br>Train: 8093<br>Test: 2023	ANN,SVM	ANN: MAE=0.0202 R^2=0.9836(test sets)	[99]
T, P, molecular weight	298-343K<br>96-28906 mbar<br>Total: 430	MLFNN	RMSE=9.06-E0 3 R^2=0.995	[100]
Model 1: T, P, Mw<br>Model 2: T, P, Mw, Ether groups in ILs, Number of carbons in hydrocarbon chain,	Total: 430<br>11 ILs.	ANN<br>5-18-1	Model 2: R^2 = 0.995, MSE = 8.23E-05	[101]
T, P	501-690K<br>16600-19588 kPa<br>Total: 548<br>Train: 438<br>Test: 110	ANN, MLP, LSSVM, RBF, ANFIS, CFNN, GRNN	GFNN: AARD=6.88% MSE=8×10^{-4} R^2 = 0.9881(total sets)	[102]
Molecular graph	Total: 414972	MP-MCTS, RNN	MP-MCTS is best model.	[103]

T, P, Tc, Pc, ω	271.11-453.15 K<br>0.0089-100.12 MPa<br>Total: 4726<br>Train: 80%<br>Test: 20%	GMDH	R^2 = 0.92	[104]
Molecular weight (Mw), Tc, Pc, w	298-373.15 K<br>0.01-100.12 MPa<br>Total: 744<br>Train: 80%<br>Test: 20%	MLP, GEP	MLP:<br>R^2 = 0.9965<br>RMSE = 0.0116	[97]
T, P, Mw of IL & gas, Pc of IL & gas, Tc of IL & gas, kinetic diameter of gas,acentric factor of IL & gas,	293.1-543.15 K<br>0.1-971 bars<br>Total: 1191<br>26 ILs	Hybrid ANFIS, MLP-ANN, CSA-LSSVM, PSO-ANFIS	MLP-ANN:<br>R^2 = 0.9935<br>AARD% = 10.5<br>MSE = 0.0003	[105]
T, P,Mw, Tc, Pc	278.12-453.15 K<br>0-1001.2 bars<br>Total: 4107<br>Train: 3286<br>Test: 821	LR RF, KNN, SVM, MLP, DT, Bagging AdaBoost, Voting Bayesian Stacking Gradient Boosting	Stacting:<br>MAE =0.001<br>R^2=0.969	[106]
ILs molecular structure, thermodynamic properties	298 or 303 K<br>1 or 2 MPa<br>Total: 180<br>Train: 166<br>Test: 14	BPNN	R^2=0.9928<br>RMSE=0.0032	[107]
T, P	243.2-453.15 K<br>0.00798-499.9 bars<br>Total: 10117<br>Train: 8094<br>Test: 2023	GIN, GAT, GCN, SVM, RF, XGBoost, MLP	GIN:<br>MAE=0.0137<br>R^2=0.9884	[108]
chemical structure	Total: 8167<br>Train: 7350<br>Test: 817	RF	RF: R^2=0.728<br>MAE=0.277	[109]
density, molecular weight, number of atoms and bonds,water content	278.12-450.49 K<br>0.0001-1001.2 bars<br>Total: 4397<br>Train: 3078<br>Test: 660<br>Validate: 659	FFNN, RBFNN, SVM	FFNN:<br>R^2=0.952<br>RMSE = 0.071<br>MAPE = 0.544	[110]

T, P, Tc, ω, Pc, Zc	273.15-449.41 K<br>0.010-100.120 MPa<br>Total: 2099<br>Train: 1890<br>Test: 105<br>Validate: 104	MLP	AARD%= 2.29%<br>AAD%= 6.74%	[111]
Model 1: Tc, melting temperature, & IL molecular weight<br>Model 2: Tc & Tp, acentric factor	278.1-368.4 K<br>0.34-14596 kPa<br>Total: 1047<br>26 ILs	ANFIS	Model 2: R^2 = 0.995<br>Model 1: R^2 = 0.95	[112]
T, P, Tc, ω, Pc, mole fraction of water	248-373 K<br>0.7-10004 kPa<br>Total: 546<br>6 ILs	SVM, ANN, LSSVM	ANN:<br>R^2 = 0.9965;<br>RMSE = 0.0104	[113]
T, Mw, Molality, CO_2 Activity coefficient	298-333.15 K<br>1-50 bars<br>Total: 2592<br>18 ILs	SVM, ANN, GPR	GPR: R^2 = 1,<br>RMSE = 0.0002<br>ANN: R^2 = 1,<br>RMSE = 0.001	[114]
T, P, Mw, Tc, Pc	292.65-450.49 K<br>0.0098-72.24 MPa<br>Total: 1517<br>Train: 1214<br>Test: 303	SVR, PSO, GWO, SSA	PSO-SVR:<br>RMSE =0.01881<br>R^2=0.9824	[70]
38 IFs information, T, P	243.2-453.15 K<br>0.0002-499.9 bars<br>Total: 13055<br>Train: 10444<br>Test: 2611	SVM,ANN	SVM:<br>R^2=0.9855<br>MSE =0.0008(train sets)	[115]
Geometric, electronic features	313.15 or 333.15 K<br>0.1-5 MPa<br>Total: 402114<br>Train: 392114<br>Test: 10000	GPR, RFR, XGBoost	GPR:<br>RMSE < 1.5<br>R^2 > 0.90<br>MAE < 0.13	[116]
T, P, Anionic and cationic structures of ILs	243.2-453.15 K<br>0.826-49990 kPa<br>Total: 9224<br>Train: 80%<br>Test: 20%	MLP, RF, SVM	MLP：<br>R^2=0.9873<br>MSE=0.0007	[117]
Chemical structure, TDU, oxygen-to-metal ratio, Molar fraction of composites	Total: 941<br>Train: 80%<br>Test: 20%	RF, XGB	XGB: R^2 = 0.71-0.87<br>RMSE= 0.33-0.55	[118]
IL loading ratio, PLD, LCD, Sacc, φ and Vfree	Total: 15140<br>Train: 80%<br>Test: 20%	CatBoost, XGBoost	CatBoost:<br>R^2= 0.990-0.949<br>MAE<1	[119]

Molecular structure	Total: 10116<br>Train: 75%<br>Test: 25%	SDVAE, DeepFM, GBPSO	MSE=0.00713	[120]
